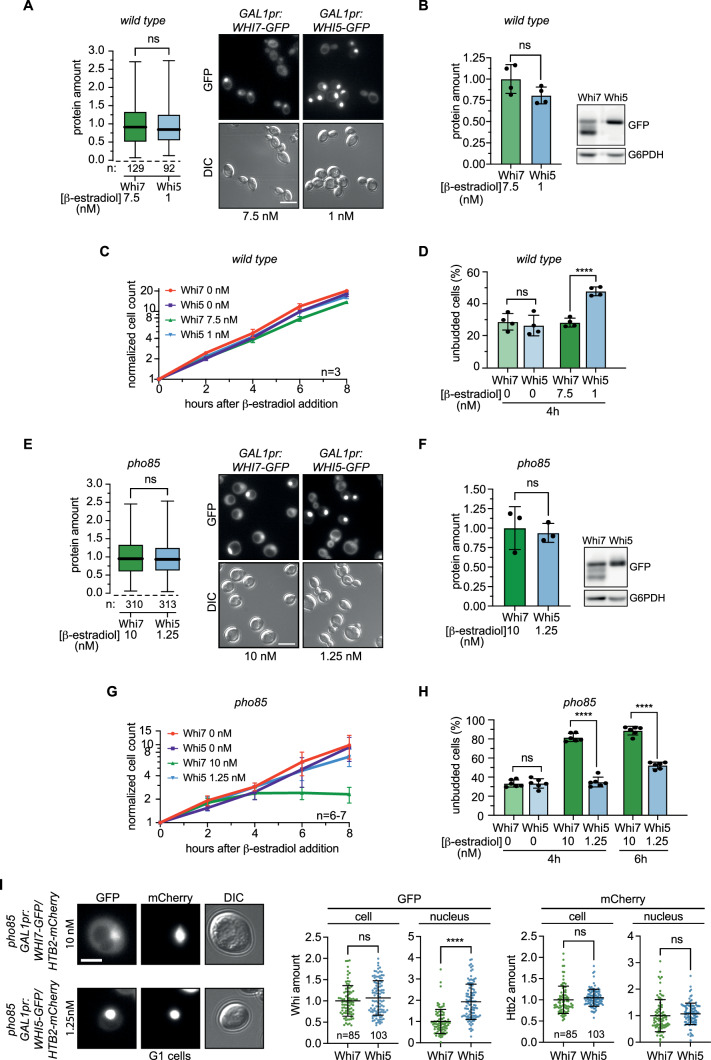# Author Correction: The CDK Pho85 inhibits Whi7 Start repressor to promote cell cycle entry in budding yeast

**DOI:** 10.1038/s44319-026-00878-2

**Published:** 2026-07-20

**Authors:** Cristina Ros-Carrero, Mihai Spiridon-Bodi, J Carlos Igual, Mercè Gomar-Alba

**Affiliations:** Institut de Biotecnologia i Biomedicina (BIOTECMED) and Departament de Bioquímica i Biologia Molecular, Universitat de València, 46100 Burjassot, Spain

## Abstract

**Figure:**
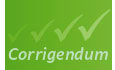

**Correction to:** *EMBO Reports* (2024) 25:745–769. 10.1038/s44319-023-00049-7 | Published online 17 January 2024

In the published version of this article, Fig. 5E contained a blank panel due to a typesetting error introduced during the conversion of figures for production. The GFP image for the GAL1pr:WHI5-GFP strain was inadvertently omitted. The error is now corrected.

This error does not affect the results, discussion, or conclusions of the article.

**Figure 5 is incorrect**

**Figure:**
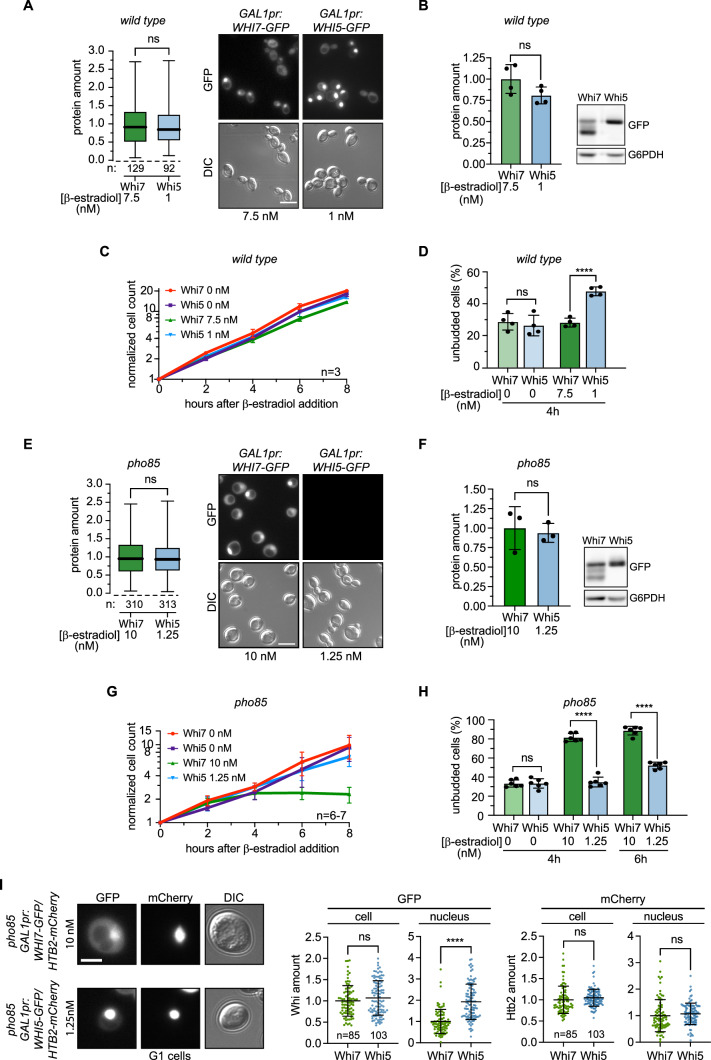

**Figure 5 is corrected**

**Figure:**