# Supplementary material for: The CDK Pho85 inhibits Whi7 Start repressor to promote cell cycle entry in budding yeast
Source: EMBO Rep. 2024 Jan 17;25(2):18. doi: 10.1038/s44319-023-00049-7 (PMC10897450; doi:10.1038/s44319-023-00049-7)
Supplement: Supplementary file 1 — Appendix [file 44319_2023_49_MOESM1_ESM.pdf]

Appendix

The CDK Pho85 inhibits Whi7 Start repressor to promote cell cycle entry in budding yeast

Cristina Ros-Carrero *et al.*

Table of contents

Appendix Figure S1 **2**

Appendix Figure S2 **3**

Appendix Table S1 **4**

Appendix Table S2 **5**

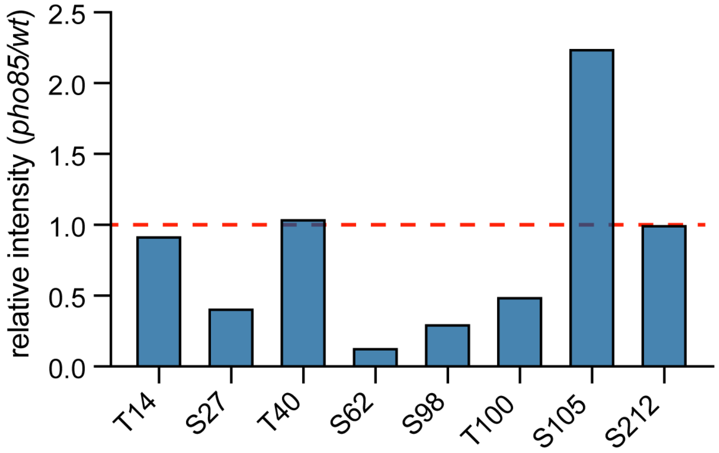

Appendix Figure S1. Several CDK consensus sites show a reduced relative phosphorylation in the *pho85* mutant. In an approximated comparison of the relative abundance of phosphorylated CDK sites in wild type and *pho85* mutant cells, we calculated in each sample the percentage of the MaxLFQ intensity for each phosphorylated CDK site relative to the total intensity of all the phosphorylated non-CDK sites in the sample. The ratio of the average of *pho85* against wild type strains for the common CDK sites is represented.

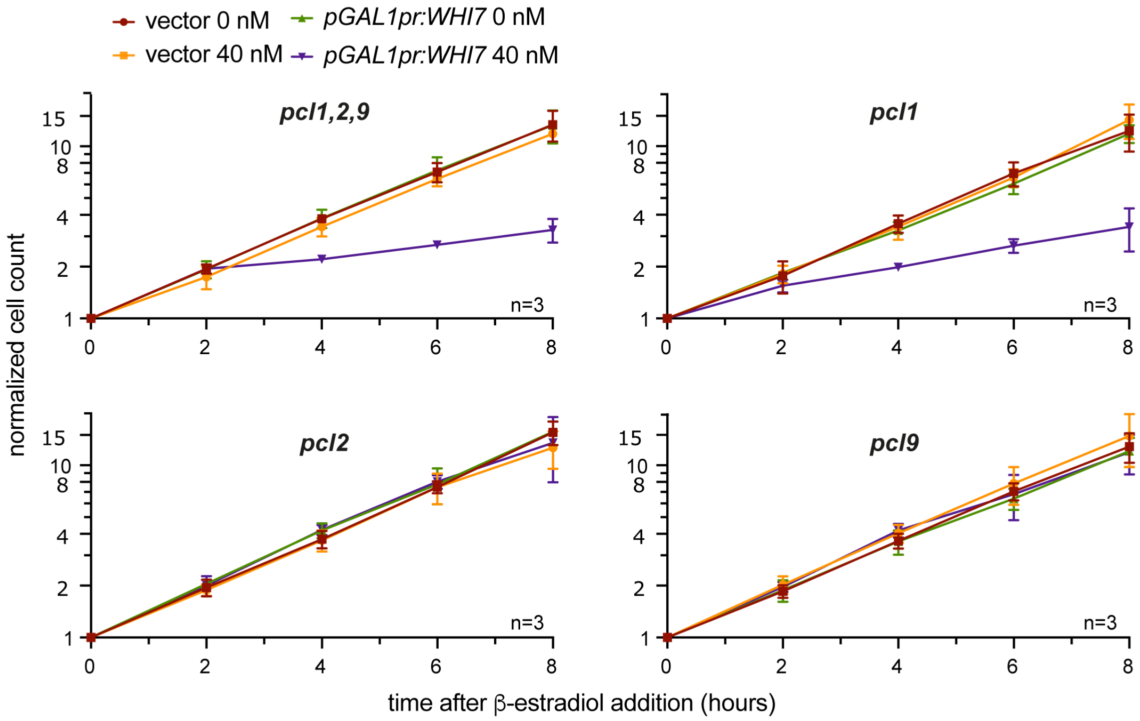

Appendix Figure S2. *WHI7* overexpression causes growth defects in the absence of Pcl1 cyclin. Exponentially growing cells of *pcl1,2,9* (JCY2626), *pcl1* (JCY2831), *pcl2* (JCY2832), or *pcl9* (JCY2833) transformed with an empty vector or the p*GAL1pr:WHI7-HA* plasmid were incubated in the presence of 0 nM or 40 nM β-estradiol (to induce overexpression of *WHI7*) and the increase in cell number over time was analyzed. Cell number was normalized to 1.

Appendix Table S1. Whi7 phosphorylation sites identified in each biological replicate.

	**wild type**			***pho85***		
	replicate 1	replicate 2	replicate 3	replicate 1	replicate 2	replicate 3
**T5**		X	X			
**T14**	X	X	X	X	X	X
**S17**		X				
**S27**	X	X	X	X		X
**T32**	X	X	X			
**S34**	X	X				X
**S36**	X	X	X	X		X
**T40**	X	X	X	X	X	X
**S56**	X	X		X		
**S62**	X	X	X	X	X	X
**T70**	X				X	
**Y71**		X	X			
**Y73**	X	X		X		X
**T84**	X	X	X	X		
**S90**	X					
**S98**	X	X	X	X	X	X
**T100**	X	X	X	X	X	X
**S105**	X	X	X	X		X
**T107**		X				
**T151**	X		X	X	X	
**S158**	X		X		X	X
**S172**		X	X	X	X	X
**S176**			X			
**S186**		X	X			
**S187**		X		X		
**S203**		X				
**S212**	X	X	X	X	X	X
**T222**	X	X	X	X	X	X
**S224**	X	X	X	X	X	X
**T225**	X	X	X	X	X	
**S229**	X	X	X	X	X	

Appendix Table S2. *Saccharomyces cerevisiae* strains used in this work

Name	Strain	Genotype	Source
W303-1a	*wild type (WT)*	*MATa ade2-1 trp1-1 leu2-3,112 his3-11,15 ura3-52 can1-100*	
JCY1539	*grr1::LEU2*	*MATa ade2-1 trp1-1 leu2-3,112 his3-11,15 ura3-52 can1-100 grr1::LEU2*	Gomar-Alba et al., 2017
JCY1728	*WHI7-HA*	*MATa ade2-1 trp1-1 leu2-3,112 his3-11,15 ura3-52 can1-100 WHI7-HA-TRP1*	Gomar-Alba et al., 2017
JCY1746	*WHI7-GFP*	*MATa ade2-1 trp1-1 leu2-3,112 his3-11,15 ura3-52 can1-100 WHI7-GFP-kanMX6*	Gomar-Alba et al., 2017
JCY1819	*whi7*	*MATa ade2-1 trp1-1 leu2-3,112 his3-11,15 ura3-52 can1-100 whi7::kanMX6*	Gomar-Alba et al., 2017
JCY1874	*whi5*	*MATa ade2-1 trp1-1 leu2-3,112 his3-11,15 ura3-52 can1-100 whi5::LEU2*	Gomar-Alba et al., 2017
JCY1976	*SWI4-myc*	*MATa ade2-1 trp1-1 leu2-3,112 his3-11,15 ura3-52 can1-100 SWI4-myc-HIS3*	Gomar-Alba et al., 2017
JCY2036	*WHI5-HA*	*MATa ade2-1 trp1-1 leu2-3,112 his3-11,15 ura3-52 can1-100 WHI5-HA-HIS3*	Gomar-Alba et al., 2017
JCY2221	*WHI7-HA pho85*	*MATa ade2-1 trp1-1 leu2-3,112 his3-11,15 ura3-52 can1-100 WHI7-HA-TRP1 pho85::kanMX6*	This study
JCY2241	*pho85*	*MATa ade2-1 trp1-1 leu2-3,112 his3-11,15 ura3-52 can1-100 pho85::kanMX6*	This study
JCY2256	*WHI7-HA pcl1 pcl2 pcl9*	*MATa ade2-1 trp1-1 leu2-3,112 his3-11,15 ura3-52 can1-100 WHI7-HA-TRP1 pcl1::kanMX6 pcl2::HIS3 pcl9::NAT*	This study
JCY2282	*WHI5-HA pho85*	*MATa ade2-1 trp1-1 leu2-3,112 his3-11,15 ura3-52 can1-100 WHI5-HA-HIS3 pho85::kanMX6*	This study
JCY2315	*pcl1 pcl2 pcl9*	*MATa ade2-1 trp1-1 leu2-3,112 his3-11,15 ura3-52 can1-100 pcl1::kanMX6 pcl2::HYG pcl9::NAT*	This study
JCY2333	*pho80*	*MATa ade2-1 trp1-1 leu2-3,112 his3-11,15 ura3-52 can1 pho80::kanMX6*	This study
JCY2335	*WHI7-HA pho80*	*MATa ade2-1 trp1-1 leu2-3,112 his3-11,15 ura3-52 can1-100 WHI7-HA-TRP1 pho80::kanMX6*	This study
JCY2337	*WHI7-HA pcl1 pcl2 pcl9 pho80*	*MATa ade2-1 trp1-1 leu2-3,112 his3-11,15 ura3-52 can1-100 WHI7-HA-TRP1 pcl1::kanMX6 pcl2::HIS3 pcl9::NAT pho80::HYG*	This study

JCY2443	*ADGEV*	*MATa ade2-1 trp1-1 leu2-3,112 his3-11,15 ura3-52 can1-100 URA3::ADGEV-URA3*	This study
JCY2446	*ADGEV GAL1:WHI7-GFP*	*MATa ade2-1 trp1-1 leu2-3,112 his3-11,15 ura3-52 can1-100 URA3::ADGEV-URA3 HIS3-GAL1:WHI7-GFP-kanMX6*	This study
JCY2448	*ADGEV GAL1:WHI5-GFP*	*MATa ade2-1 trp1-1 leu2-3,112 his3-11,15 ura3-52 can1-100 URA3::ADGEV-URA3 HIS3-GAL1:WHI5-GFP-kanMX6*	This study
JCY2475	*pho85 whi7*	*MATa ade2-1 trp1-1 leu2-3,112 his3-11,15 ura3-52 can1-100 pho85::kanMX6 whi7::HIS3*	This study
JCY2486	*ADGEV pho85*	*MATa ade2-1 trp1-1 leu2-3,112 his3-11,15 ura3-52 can1-100 pho85::kanMX6 URA3::ADGEV-URA3*	This study
JCY2495	*WHI5-mNeonGreen*	*MATa ade2-1 trp1-1 leu2-3,112 his3-11,15 ura3-52 can1-100 WHI5-mNeonGreen-URA3*	This study
JCY2497	*WHI7-mNeonGreen*	*MATa ade2-1 trp1-1 leu2-3,112 his3-11,15 ura3-52 can1-100 WHI7-mNeonGreen-URA3*	This study
JCY2499	*WHI7-mNeonGreen pho85*	*MATa ade2-1 trp1-1 leu2-3,112 his3-11,15 ura3-52 can1-100 pho85::kanMX6 WHI7-mNeonGreen-URA3*	This study
JCY2501	*WHI5-mNeonGreen pho85*	*MATa ade2-1 trp1-1 leu2-3,112 his3-11,15 ura3-52 can1-100 pho85::kanMX6 WHI5-mNeonGreen-URA3*	This study
JCY2503	*PHO4-GFP-HIS3*	*MATa ade2-1 trp1-1 leu2-3,112 his3-11,15 ura3-52 can1-100 PHO4-GFP-HIS3*	This study
JCY2505	*PHO4-GFP-HIS3 pho85*	*MATa ade2-1 trp1-1 leu2-3,112 his3-11,15 ura3-52 can1-100 PHO4-GFP-HIS3 pho85::kanMX6*	This study
JCY2538	*pho85 whi5*	*MATa ade2-1 trp1-1 leu2-3,112 his3-11,15 ura3-52 can1-100 pho85::kanMX6 whi5::LEU2*	This study
JCY2540	*pho85 whi5 whi7*	*MATa ade2-1 trp1-1 leu2-3,112 his3-11,15 ura3-52 can1-100 pho85::kanMX6 whi5::LEU2 whi7::kanMX6*	This study
JCY2553	*WHI5-GFP WHI7-myc*	*MATa ade2-1 trp1-1 leu2-3,112 his3-11,15 ura3-52 can1-100 WHI5-GFP-kanMX6 WHI7-7myc-HIS3*	This study
JCY2556	*WHI5-GFP WHI7-myc pho85*	*MATa ade2-1 trp1-1 leu2-3,112 his3-11,15 ura3-52 can1-100 WHI5-GFP-kanMX6 WHI7-7myc-HIS3 pho85::kanMX6*	This study
JCY2559	*ADGEV GAL1:WHI5-GFP pho85*	*MATa ade2-1 trp1-1 leu2-3,112 his3-11,15 ura3-52 can1-100 URA3::ADGEV-URA3 HIS3-GAL1:WHI5-GFP-kanMX6 pho85::HYG*	This study
JCY2561	*ADGEV GAL1:WHI7-GFP pho85*	*MATa ade2-1 trp1-1 leu2-3,112 his3-11,15 ura3-52 can1-100 URA3::ADGEV-URA3 HIS3-GAL1:WHI7-GFP-kanMX6 pho85::HYG*	This study

JCY2626	*ADGEV pcl1 pcl2 pcl9*	*MATa ade2-1 trp1-1 leu2-3,112 his3-11,15 ura3-52 can1-100 URA3::ADGEV-URA3 pcl1::kanMX6 pcl2::HYG pcl9::NAT*	This study
JCY2627	*ADGEV pho80*	*MATa ade2-1 trp1-1 leu2-3,112 his3-11,15 ura3-52 can1-100 URA3::ADGEV-URA3 pho80::kanMX6*	This study
JCY2652	*PHO85-TAP*	*MATa ade2-1 trp1-1 leu2-3,112 his3-11,15 ura3-52 can1-100 PHO85-TAP-TRP1*	This study
JCY2831	*ADGEV pcl1*	*MATa ade2-1 trp1-1 leu2-3,112 his3-11,15 ura3-52 can1-100 URA3::ADGEV-URA3 pcl1::kanMX6*	This study
JCY2832	*ADGEV pcl2*	*MATa ade2-1 trp1-1 leu2-3,112 his3-11,15 ura3-52 can1-100 URA3::ADGEV-URA3 pcl2::kanMX6*	This study
JCY2833	*ADGEV pcl9*	*MATa ade2-1 trp1-1 leu2-3,112 his3-11,15 ura3-52 can1-100 URA3::ADGEV-URA3 pcl9::kanMX6*	This study
JCY2941	*GAL1:WHI7-GFP ADGEV HTB2-mCherry*	*MATa ade2-1 trp1-1 leu2-3,112 his3-11,15 ura3-52 can1-100 URA3::ADGEV-URA3 HIS3-GAL1:WHI7-GFP-kanMX6 HTB2-mCherry-HYG*	This study
JCY2951	*GAL1:WHI7-GFP ADGEV HTB2-mCherry pho85*	*MATa ade2-1 trp1-1 leu2-3,112 his3-11,15 ura3-52 can1-100 URA3::ADGEV-URA3 HIS3-GAL1:WHI7-GFP-kanMX6 HTB2-mCherry-HYG pho85::NAT*	This study
JCY2978	*GAL1:WHI5-GFP ADGEV HTB2-mCherry pho85*	*MATa ade2-1 trp1-1 leu2-3,112 his3-11,15 ura3-52 can1-100 URA3::ADGEV-URA3 HIS3-GAL1:WHI5-GFP-kanMX6 HTB2-mCherry-HYG pho85::NAT*	This study
JCY2981	*HTB2-mCherry whi7*	*MATa ade2-1 trp1-1 leu2-3,112 his3-11,15 ura3-52 can1-100 HTB2-mCherry-HYG whi7::kanMX6*	This study
