# Supplementary figures and images for: The CDK Pho85 inhibits Whi7 Start repressor to promote cell cycle entry in budding yeast
Source: EMBO Rep. 2024 Jan 17;25(2):18. doi: 10.1038/s44319-023-00049-7 (PMC10897450; doi:10.1038/s44319-023-00049-7)

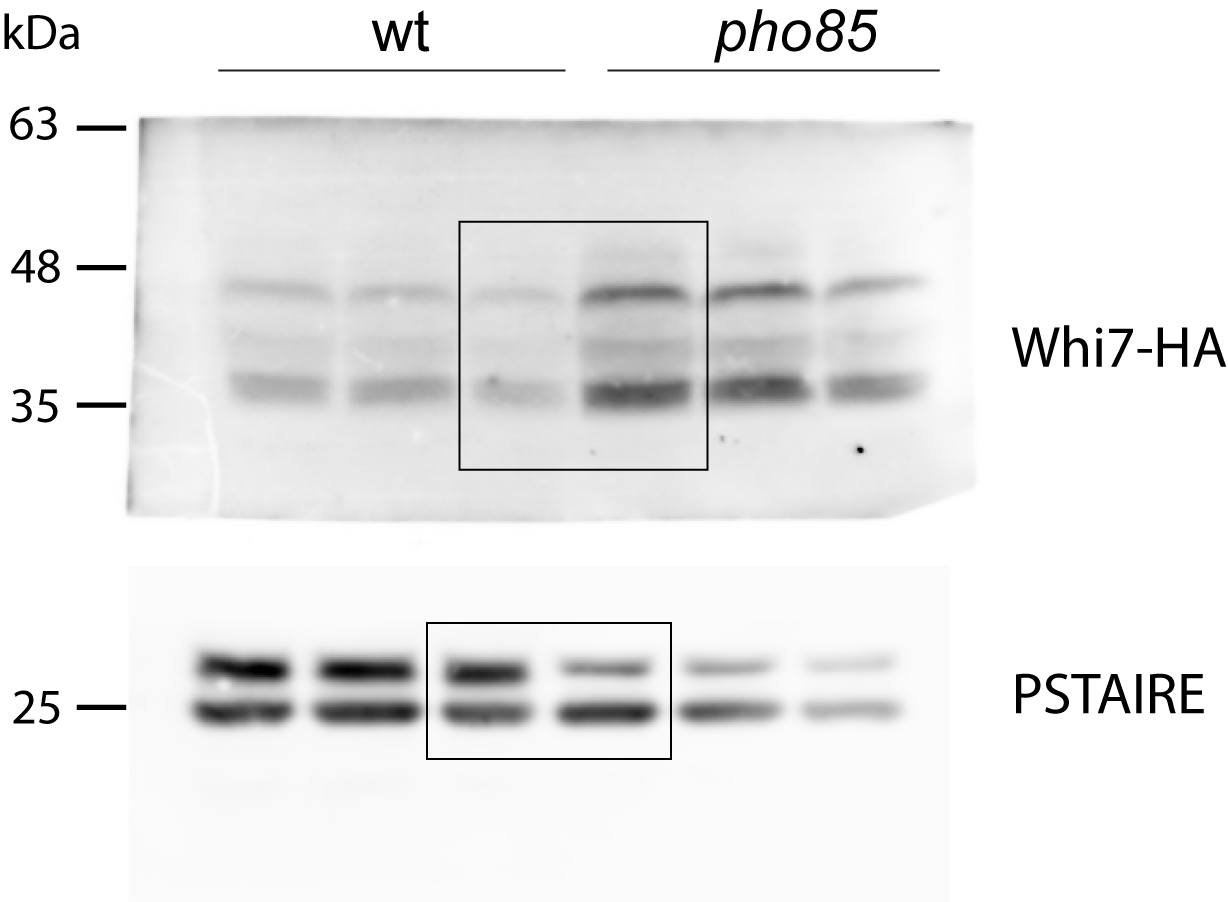

Supplement: Supplementary file 4 — Source Data Fig. 1 [file 44319_2023_49_MOESM4_ESM.zip › Figure 1/1A/WB_1A.tif]

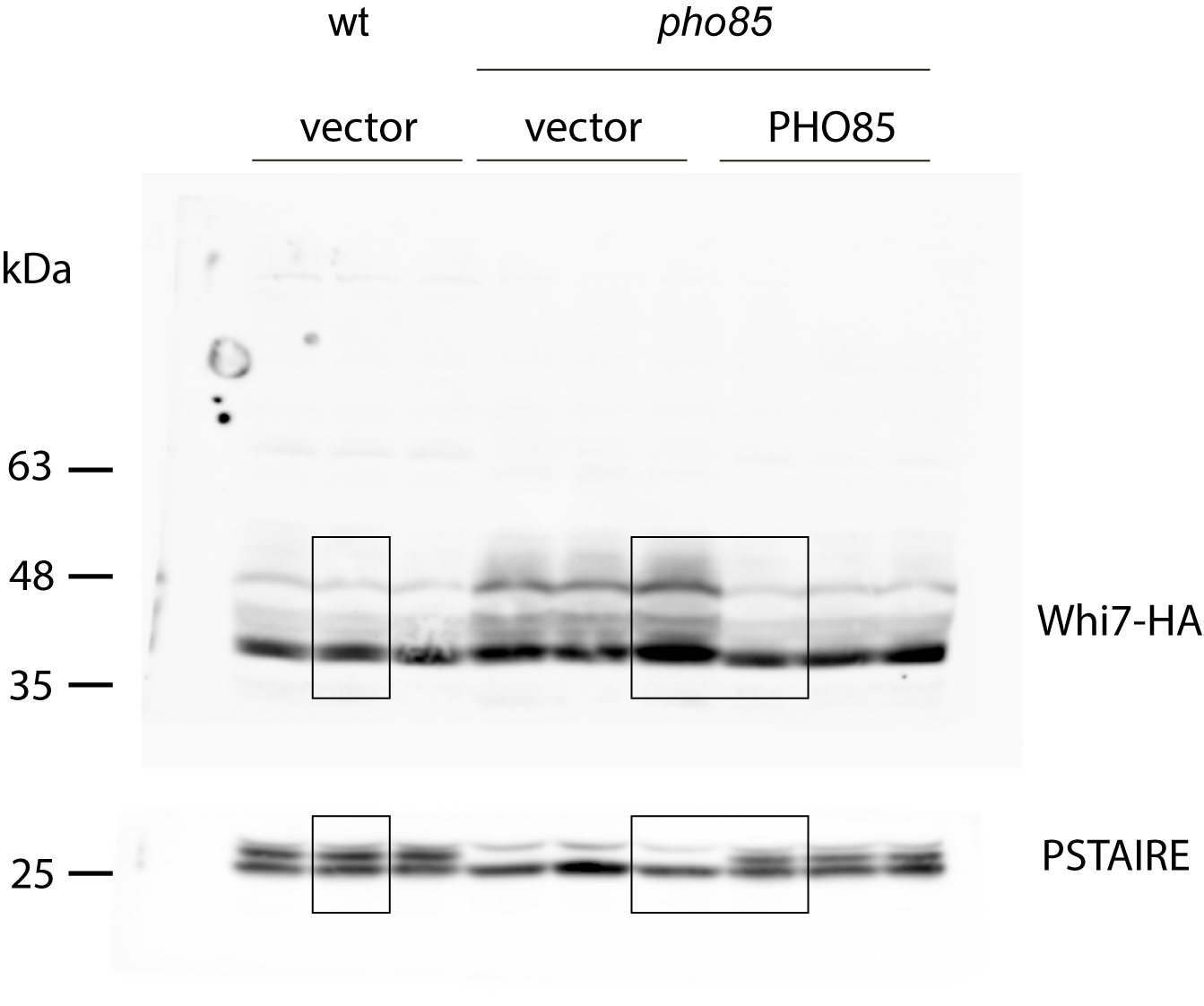

Supplement: Supplementary file 4 — Source Data Fig. 1 [file 44319_2023_49_MOESM4_ESM.zip › Figure 1/1B/WB_1B.tif]

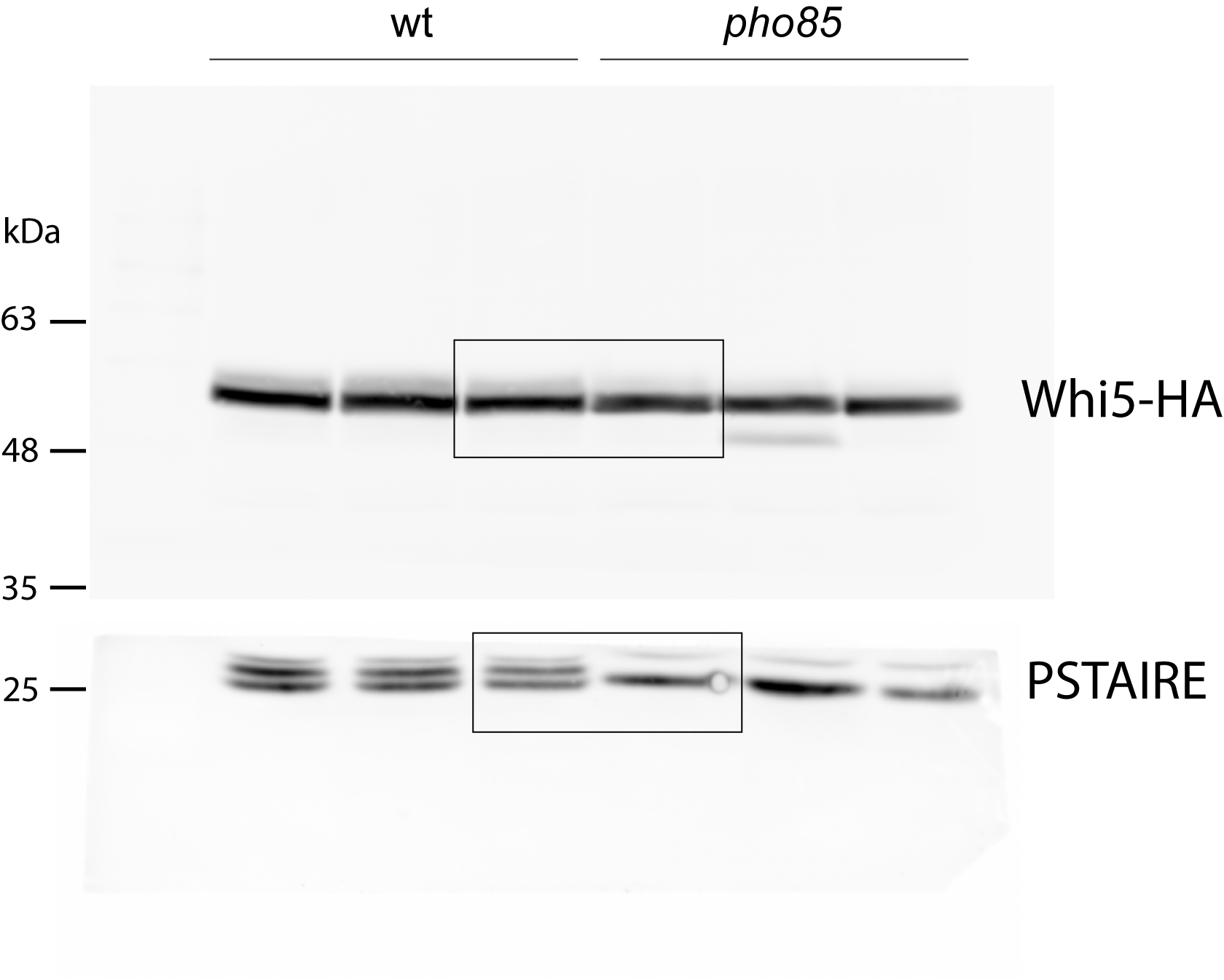

Supplement: Supplementary file 4 — Source Data Fig. 1 [file 44319_2023_49_MOESM4_ESM.zip › Figure 1/1E/WB_1E.tif]

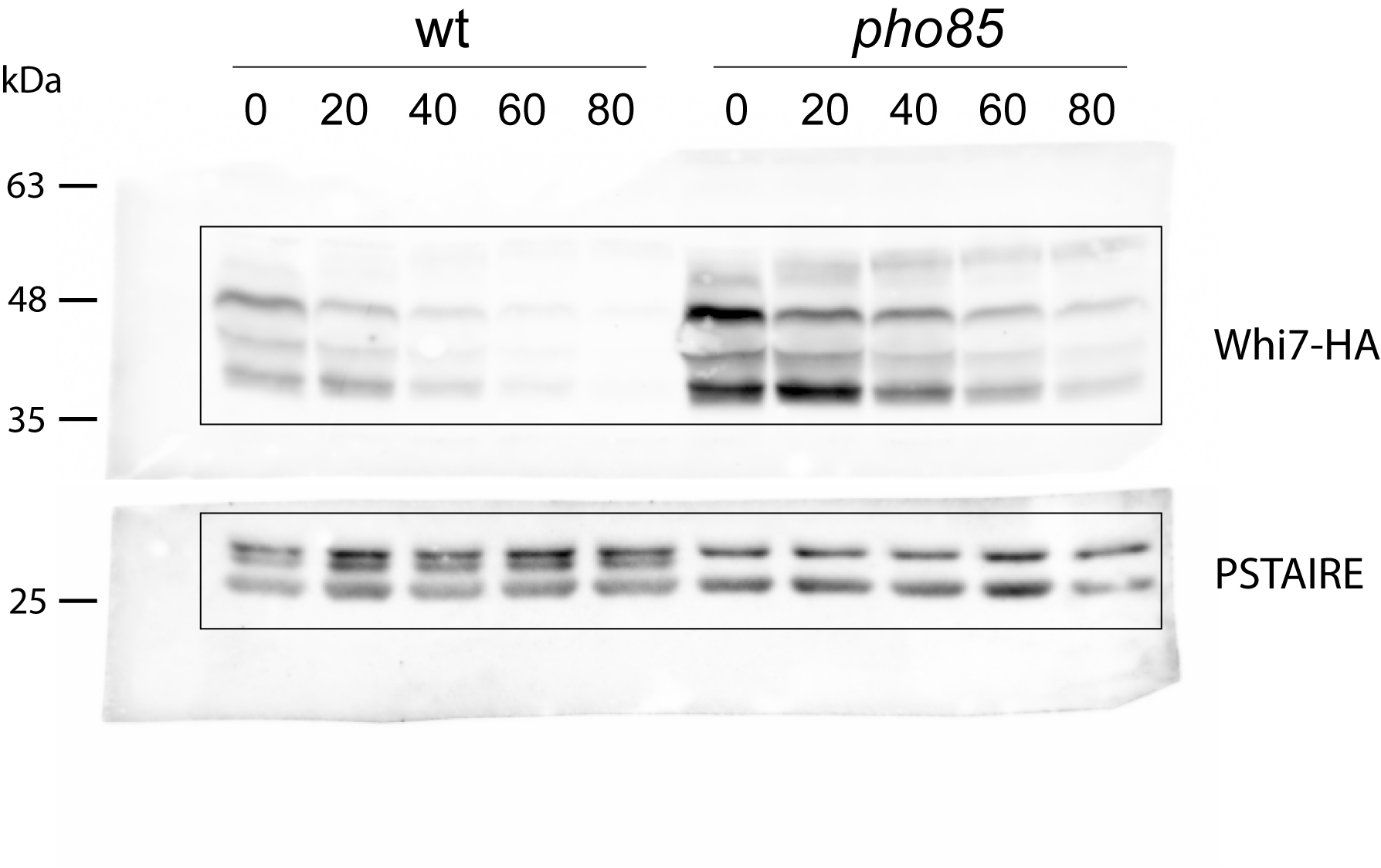

Supplement: Supplementary file 4 — Source Data Fig. 1 [file 44319_2023_49_MOESM4_ESM.zip › Figure 1/1D/WB_1D.tif]

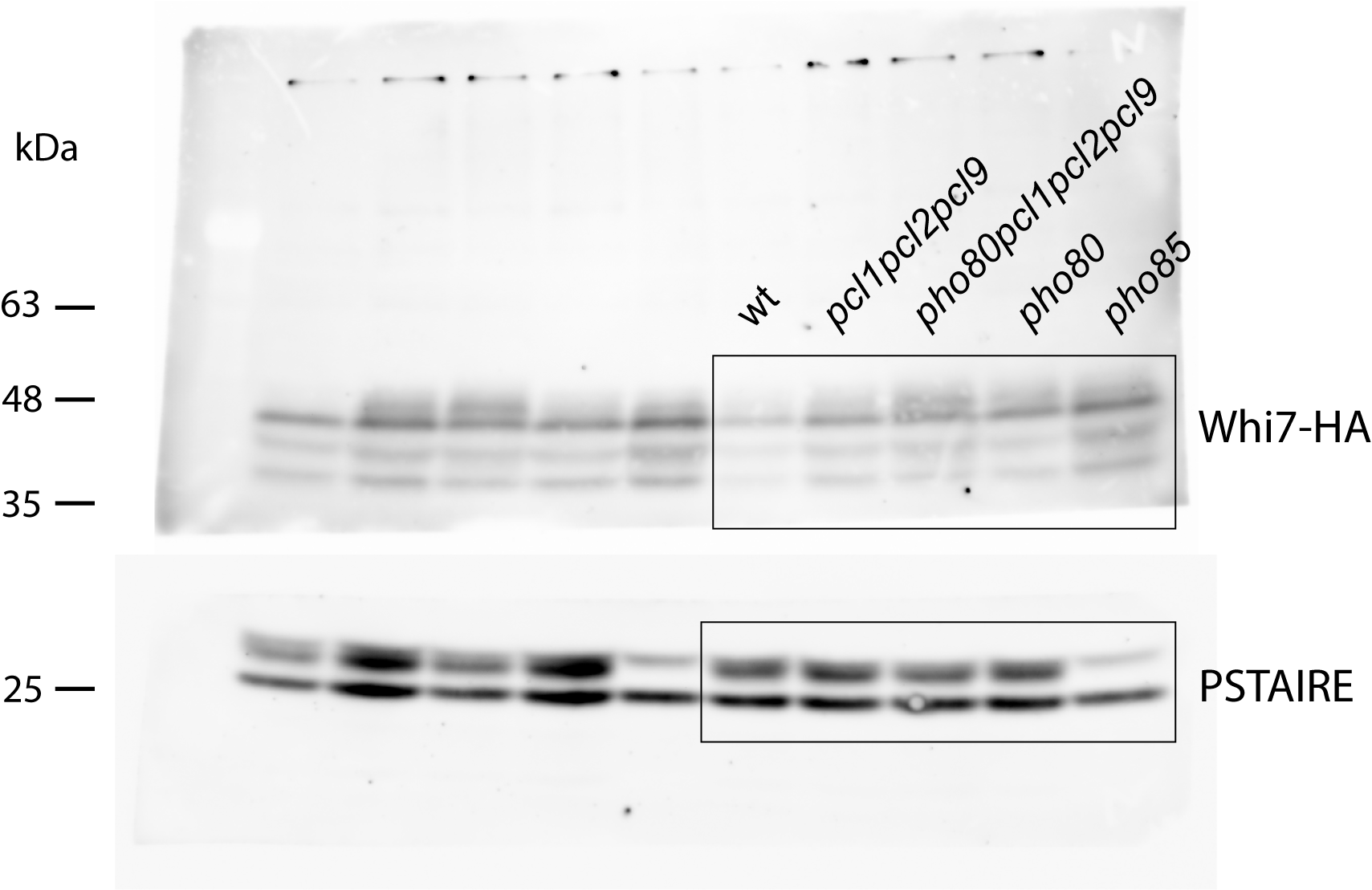

Supplement: Supplementary file 5 — Source Data Fig. 2 [file 44319_2023_49_MOESM5_ESM.zip › Figure 2/2A/WB_2A.tif]

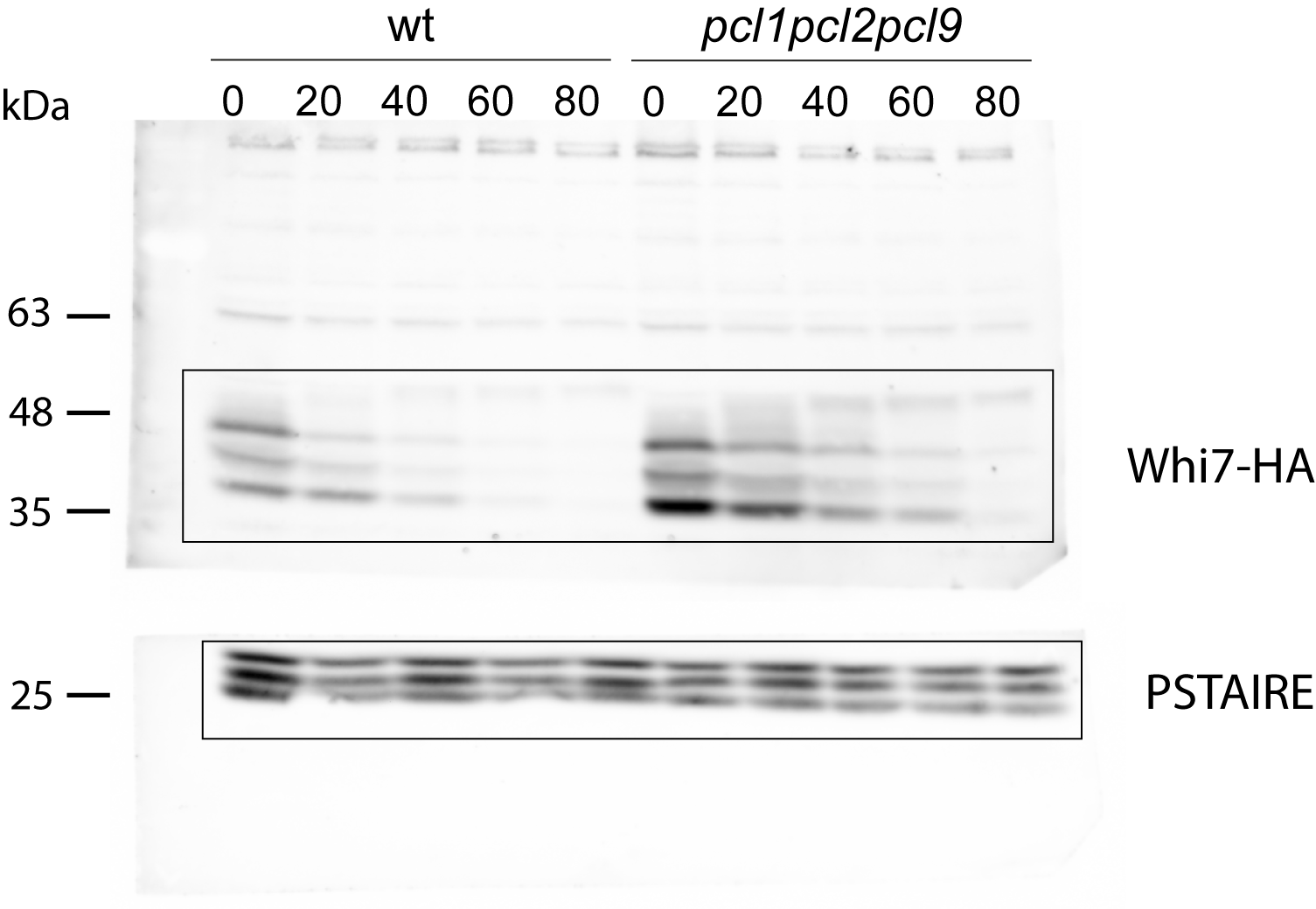

Supplement: Supplementary file 5 — Source Data Fig. 2 [file 44319_2023_49_MOESM5_ESM.zip › Figure 2/2C/WB_2C.tif]

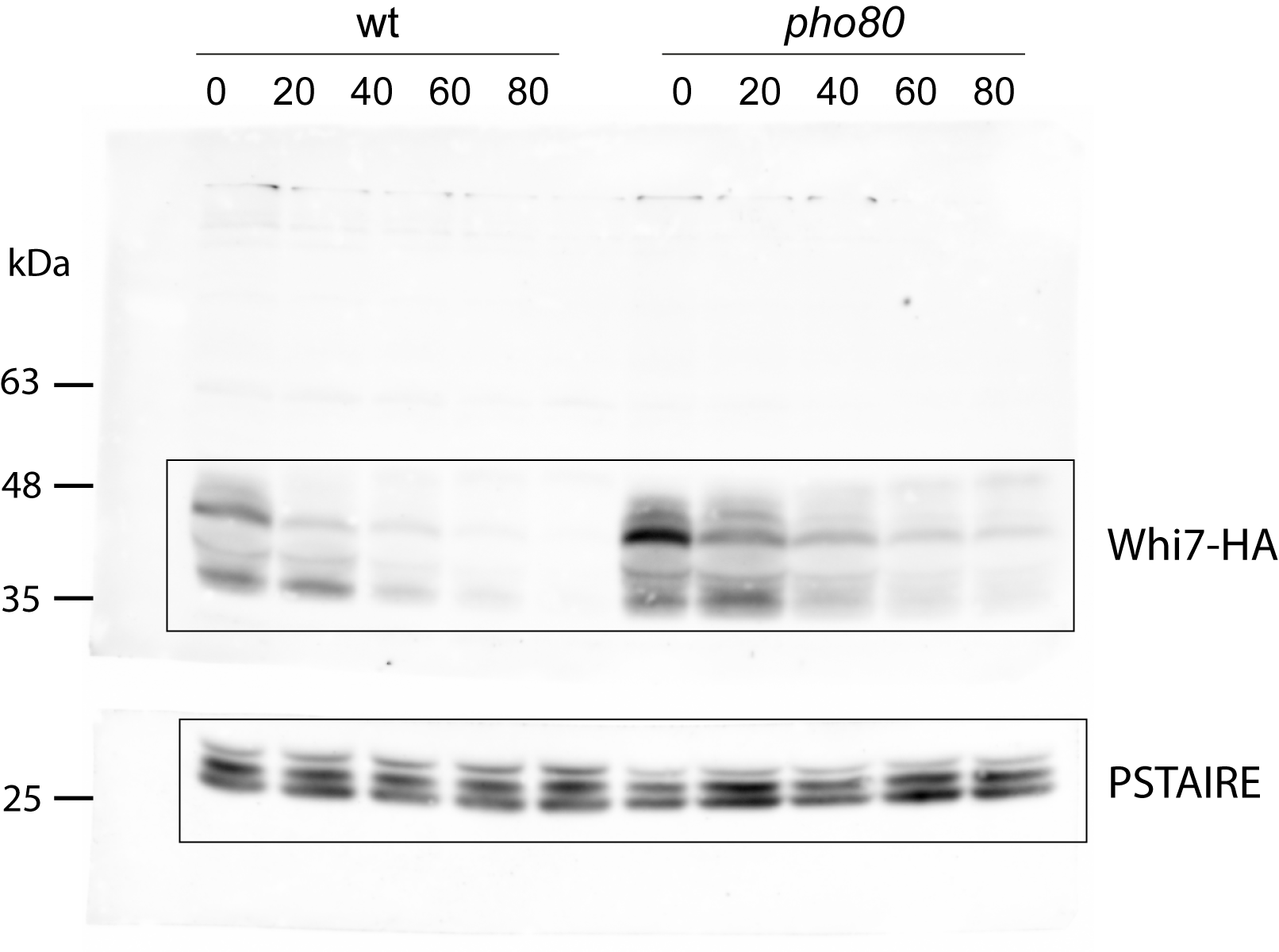

Supplement: Supplementary file 5 — Source Data Fig. 2 [file 44319_2023_49_MOESM5_ESM.zip › Figure 2/2D/WB_2D.tif]

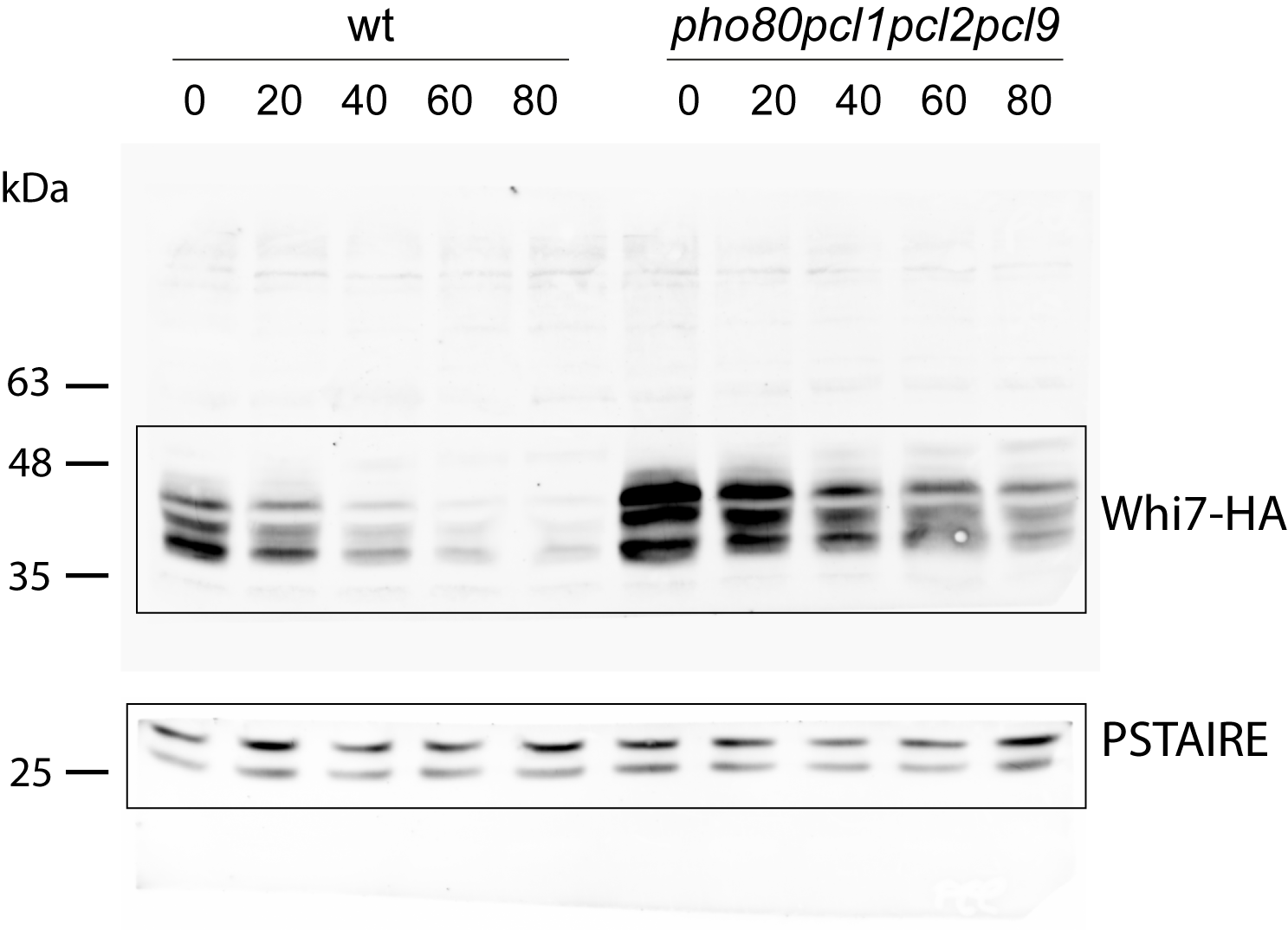

Supplement: Supplementary file 5 — Source Data Fig. 2 [file 44319_2023_49_MOESM5_ESM.zip › Figure 2/2E/WB_2E.tif]

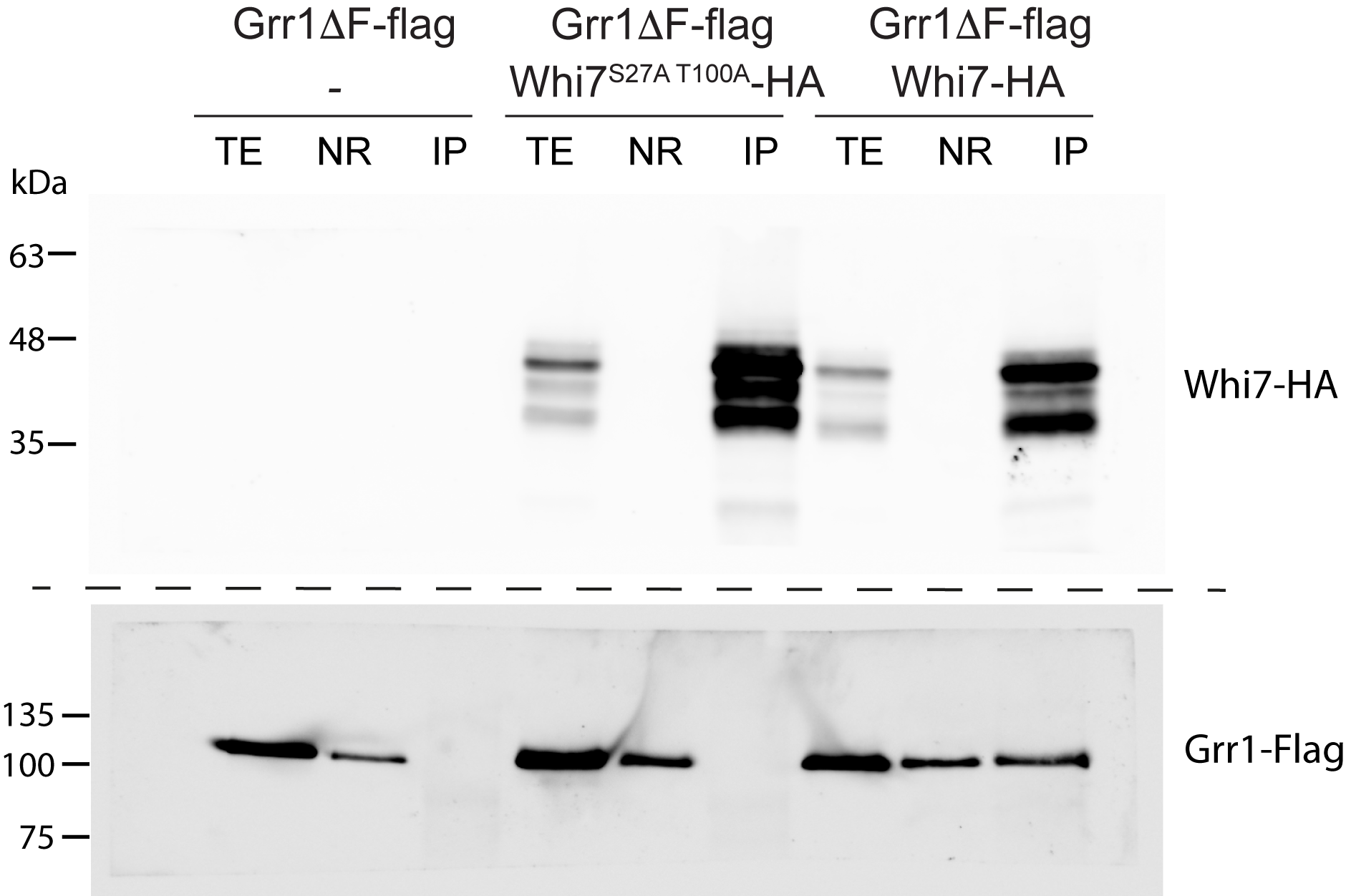

Supplement: Supplementary file 6 — Source Data Fig. 3 [file 44319_2023_49_MOESM6_ESM.zip › Figure 3/3E/WB_3E.tif]

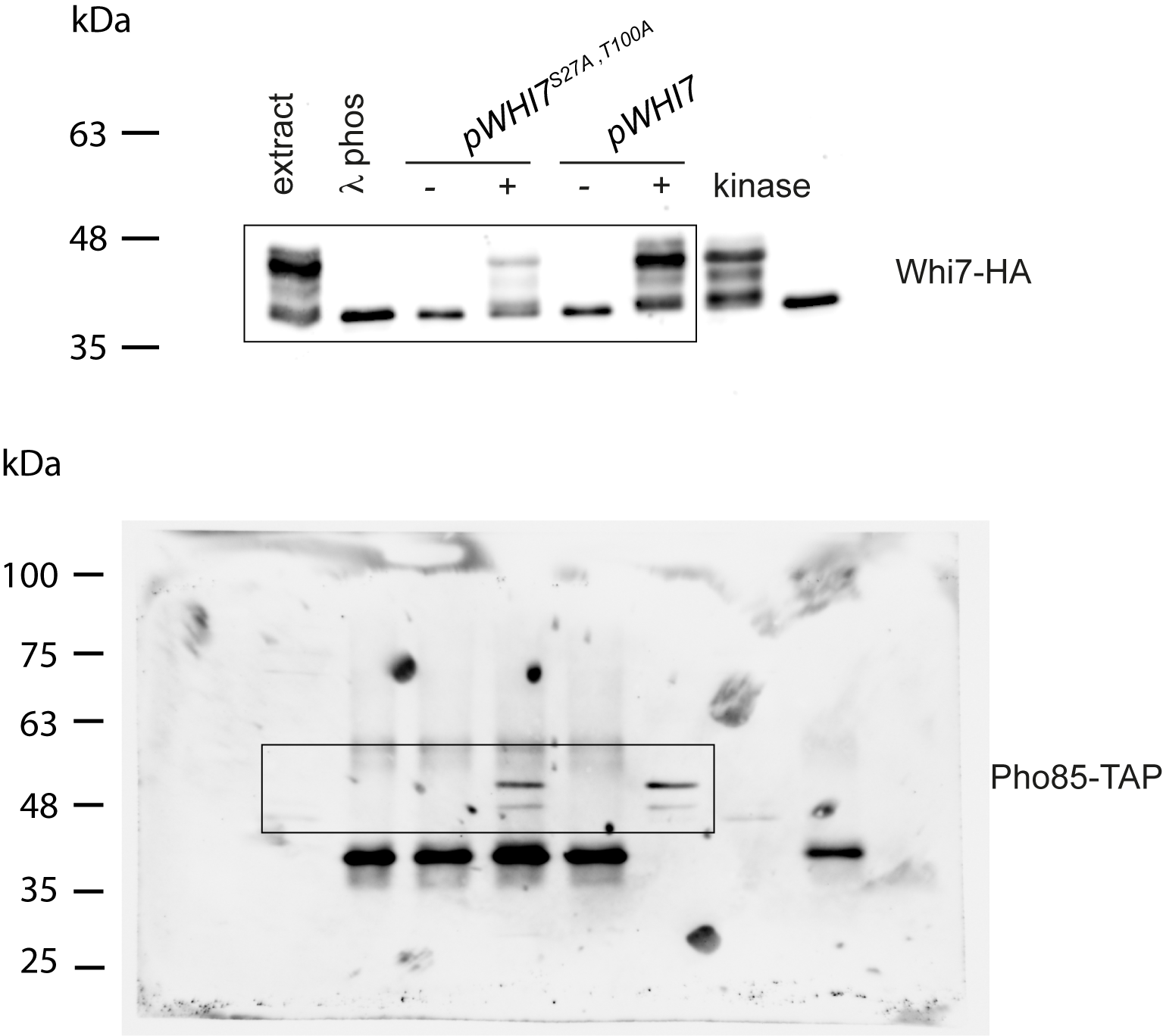

Supplement: Supplementary file 6 — Source Data Fig. 3 [file 44319_2023_49_MOESM6_ESM.zip › Figure 3/3B/WB_3B.tif]

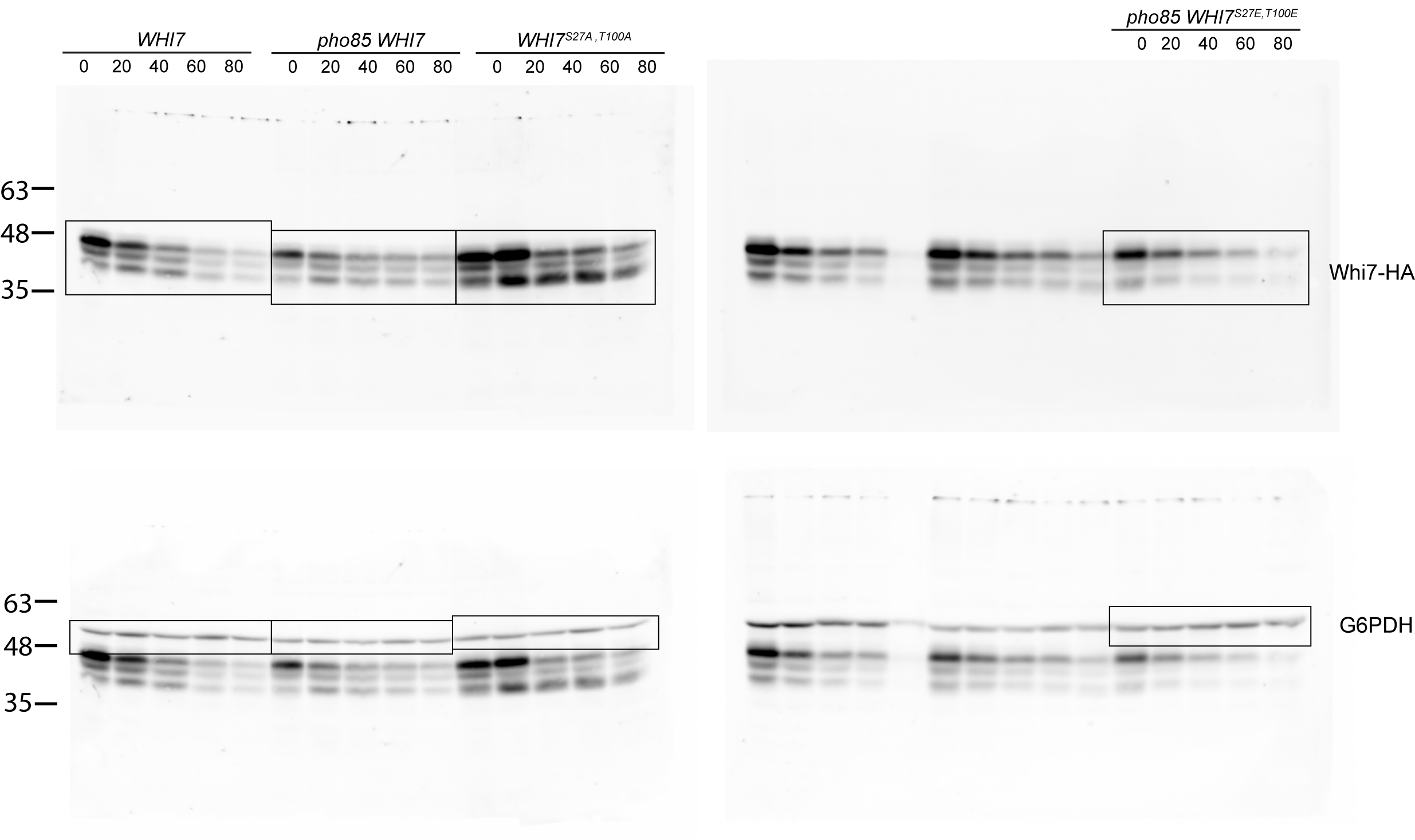

Supplement: Supplementary file 6 — Source Data Fig. 3 [file 44319_2023_49_MOESM6_ESM.zip › Figure 3/3C/WB_3C.tif]

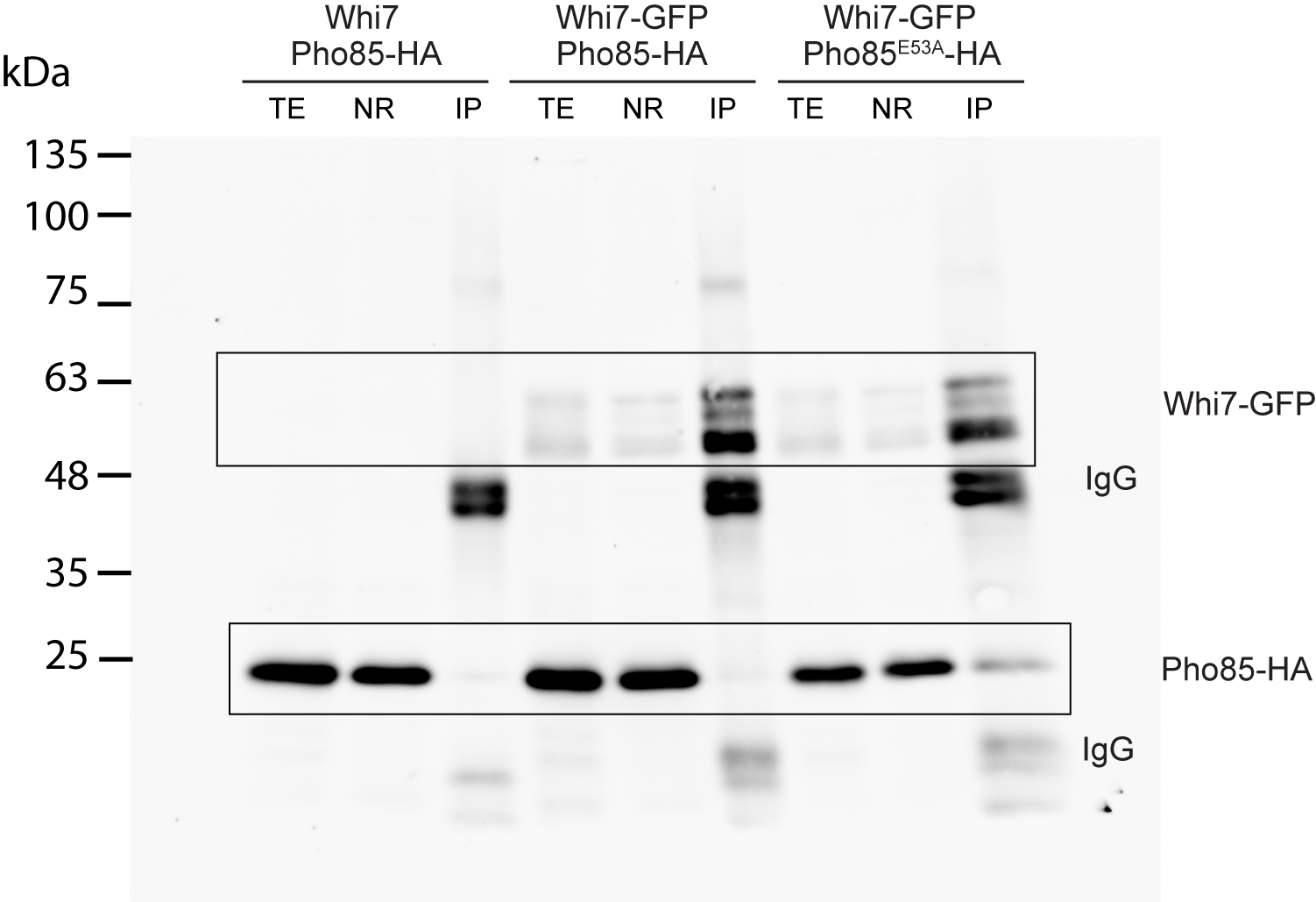

Supplement: Supplementary file 6 — Source Data Fig. 3 [file 44319_2023_49_MOESM6_ESM.zip › Figure 3/3A/WB_3A.tif]

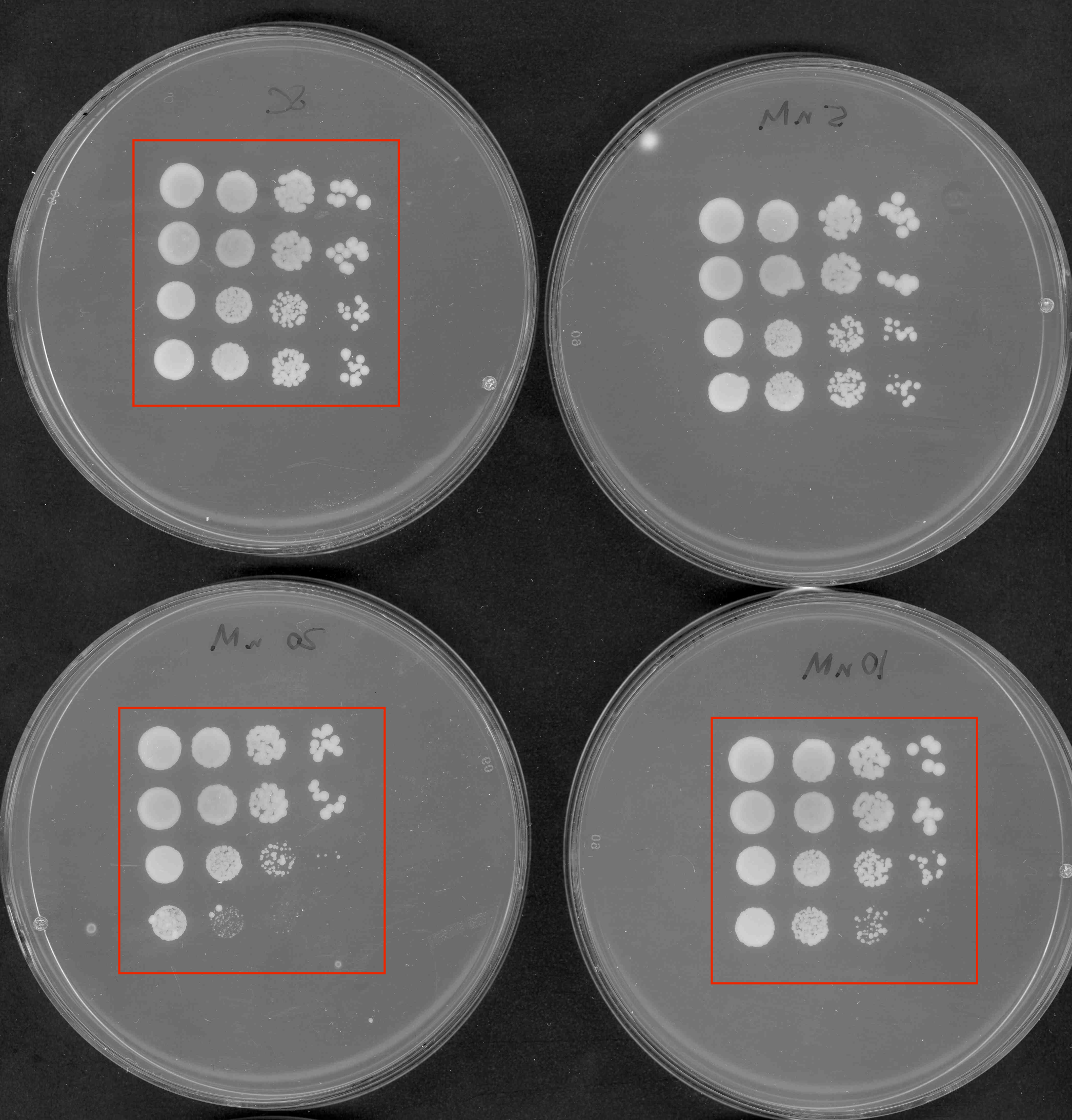

Supplement: Supplementary file 7 — Source Data Fig. 4 [file 44319_2023_49_MOESM7_ESM.zip › Figure 4/4A/4A.tif]

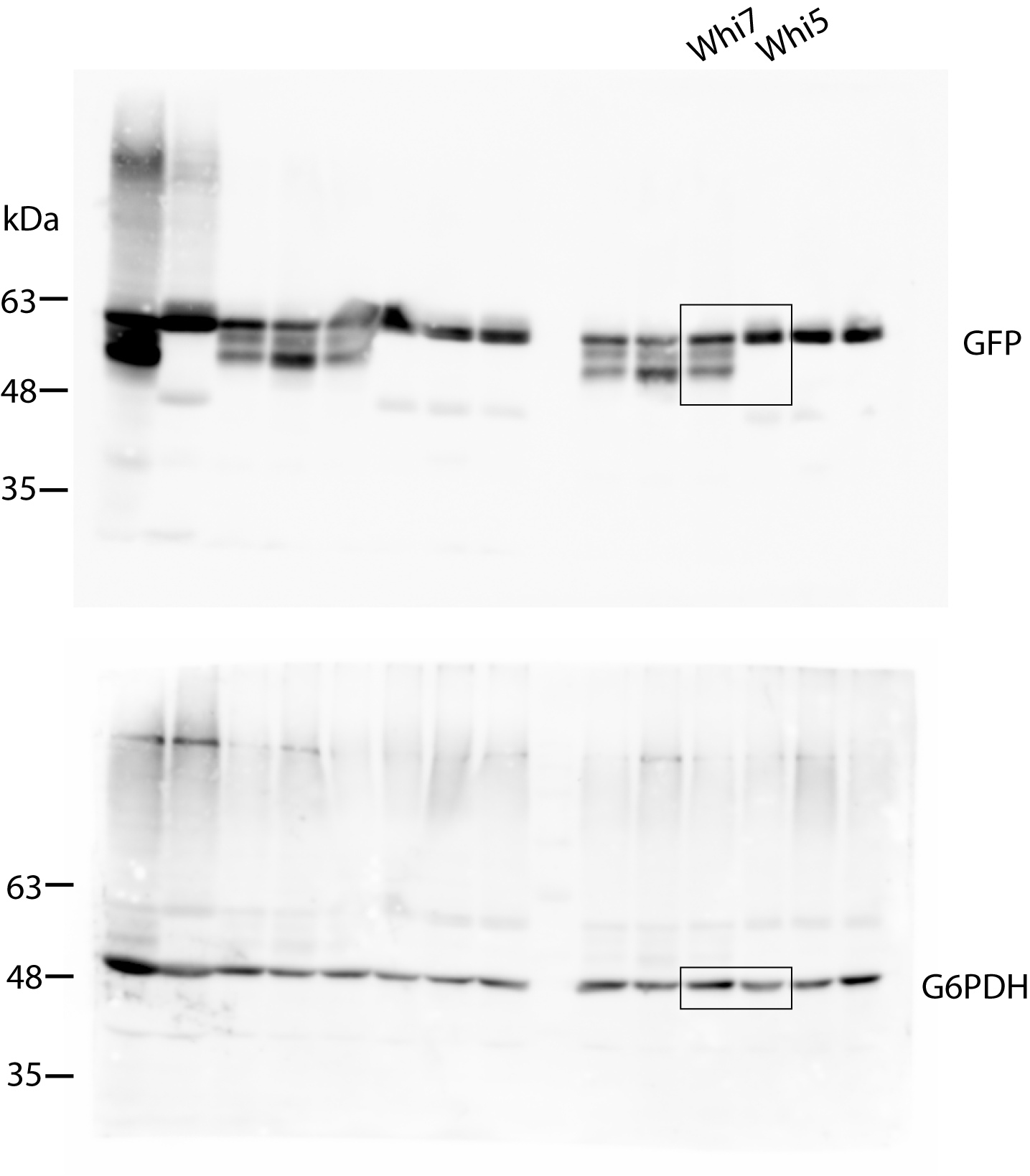

Supplement: Supplementary file 8 — Source Data Fig. 5 [file 44319_2023_49_MOESM8_ESM.zip › Figure 5/5F/WB_5F.tif]

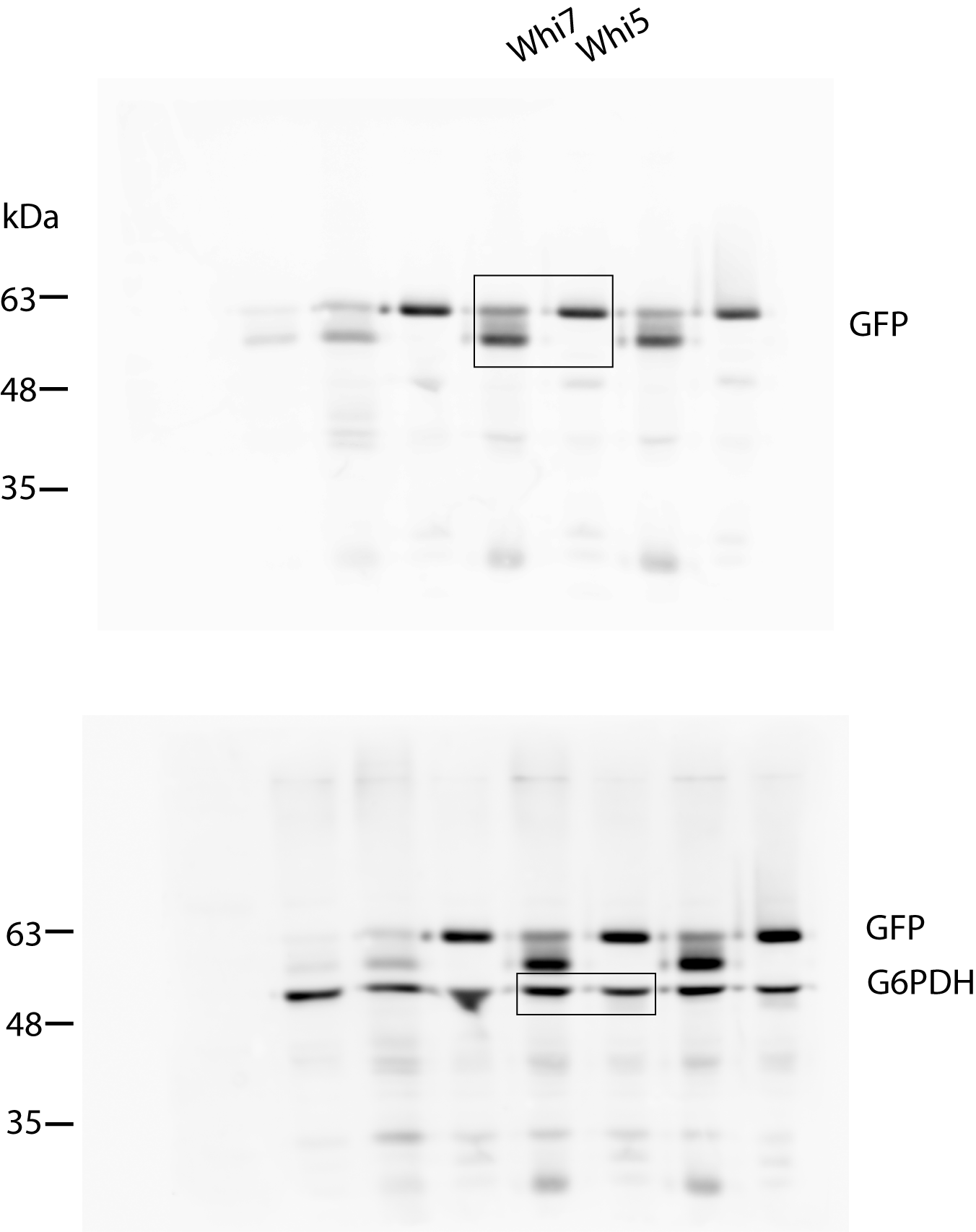

Supplement: Supplementary file 8 — Source Data Fig. 5 [file 44319_2023_49_MOESM8_ESM.zip › Figure 5/5B/WB_5B.tif]

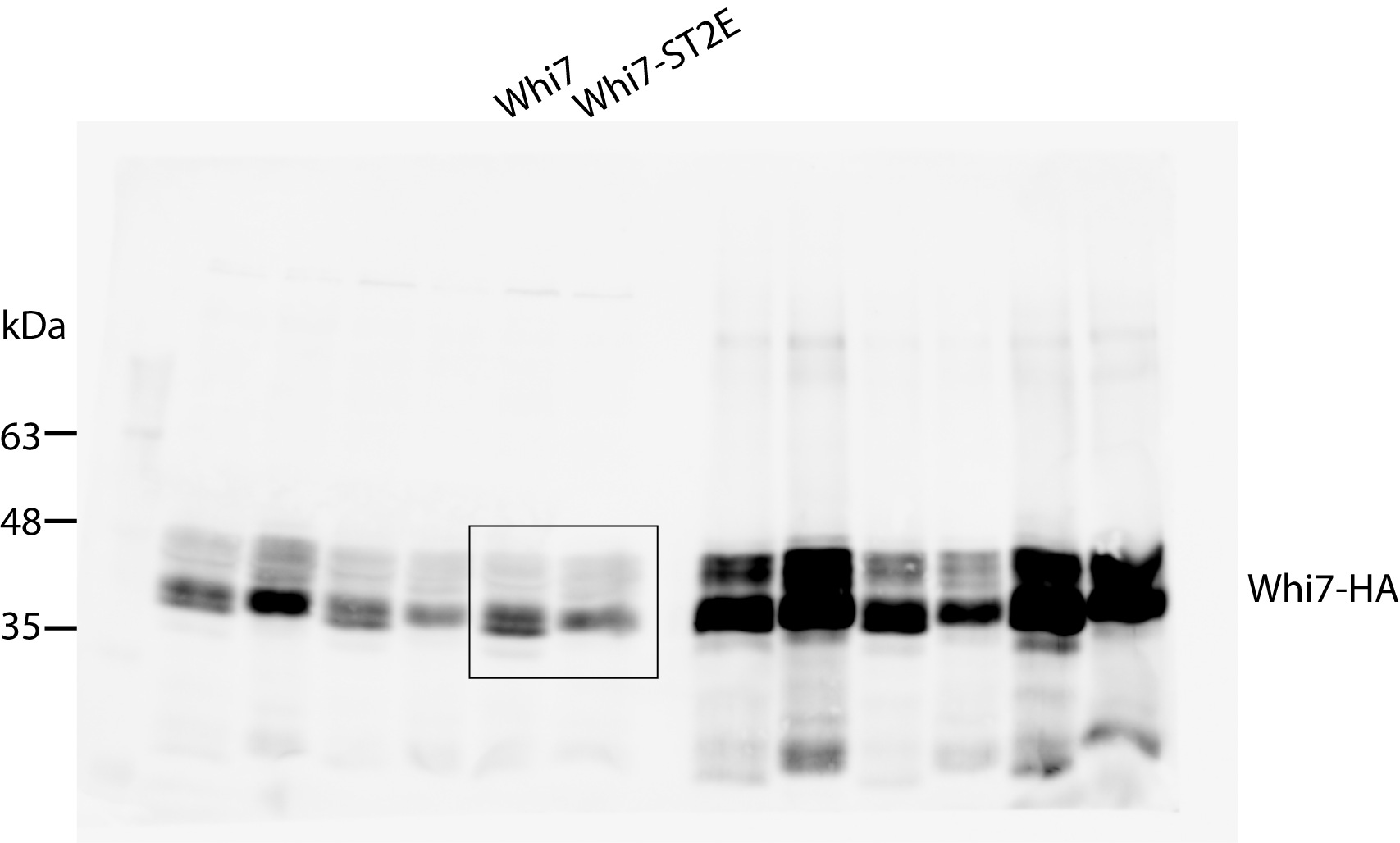

Supplement: Supplementary file 10 — Source Data Fig. 7 [file 44319_2023_49_MOESM10_ESM.zip › Figure 7/7D/WB_7D.tif]

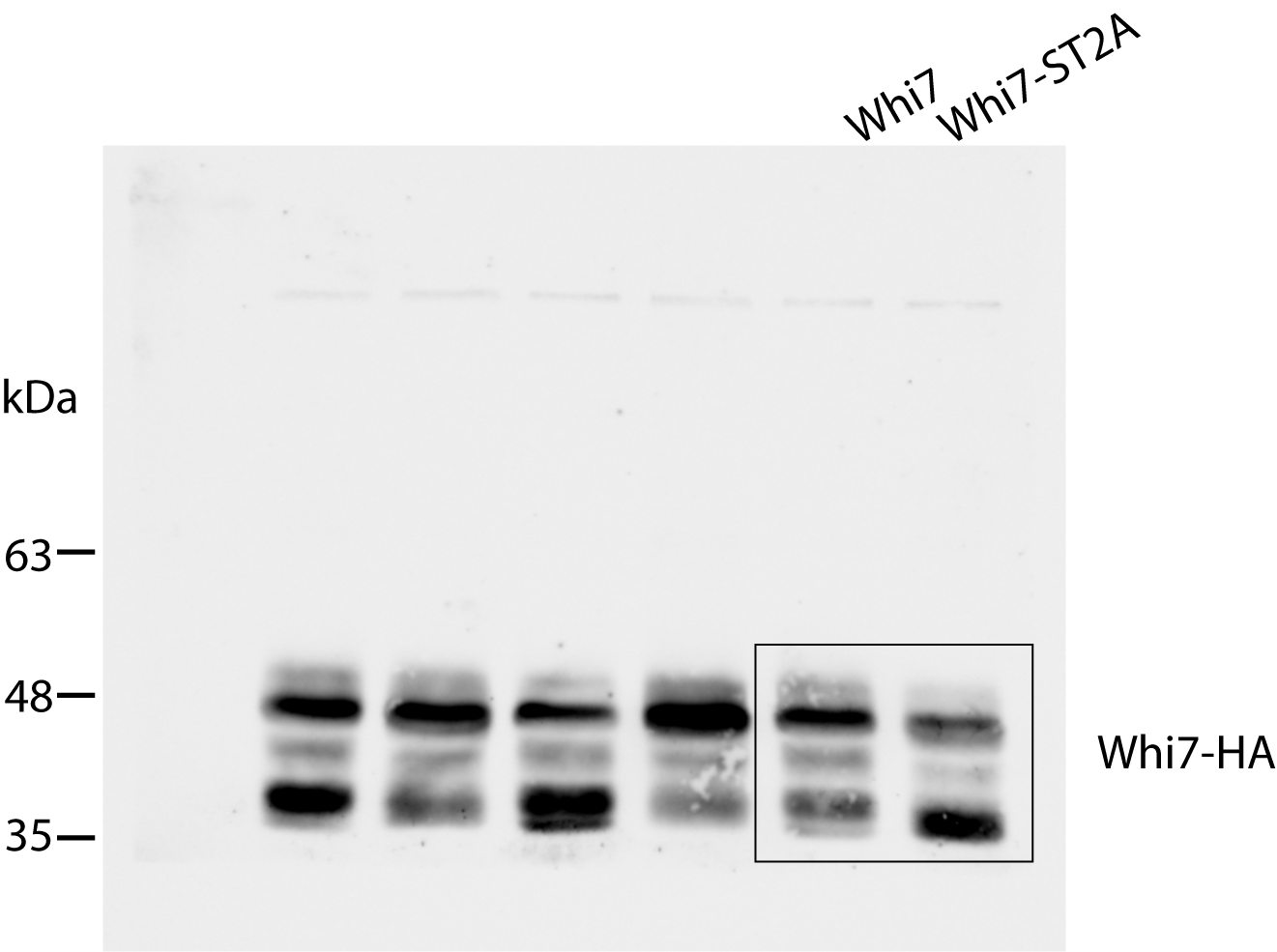

Supplement: Supplementary file 10 — Source Data Fig. 7 [file 44319_2023_49_MOESM10_ESM.zip › Figure 7/7C/WB_7C.tif]

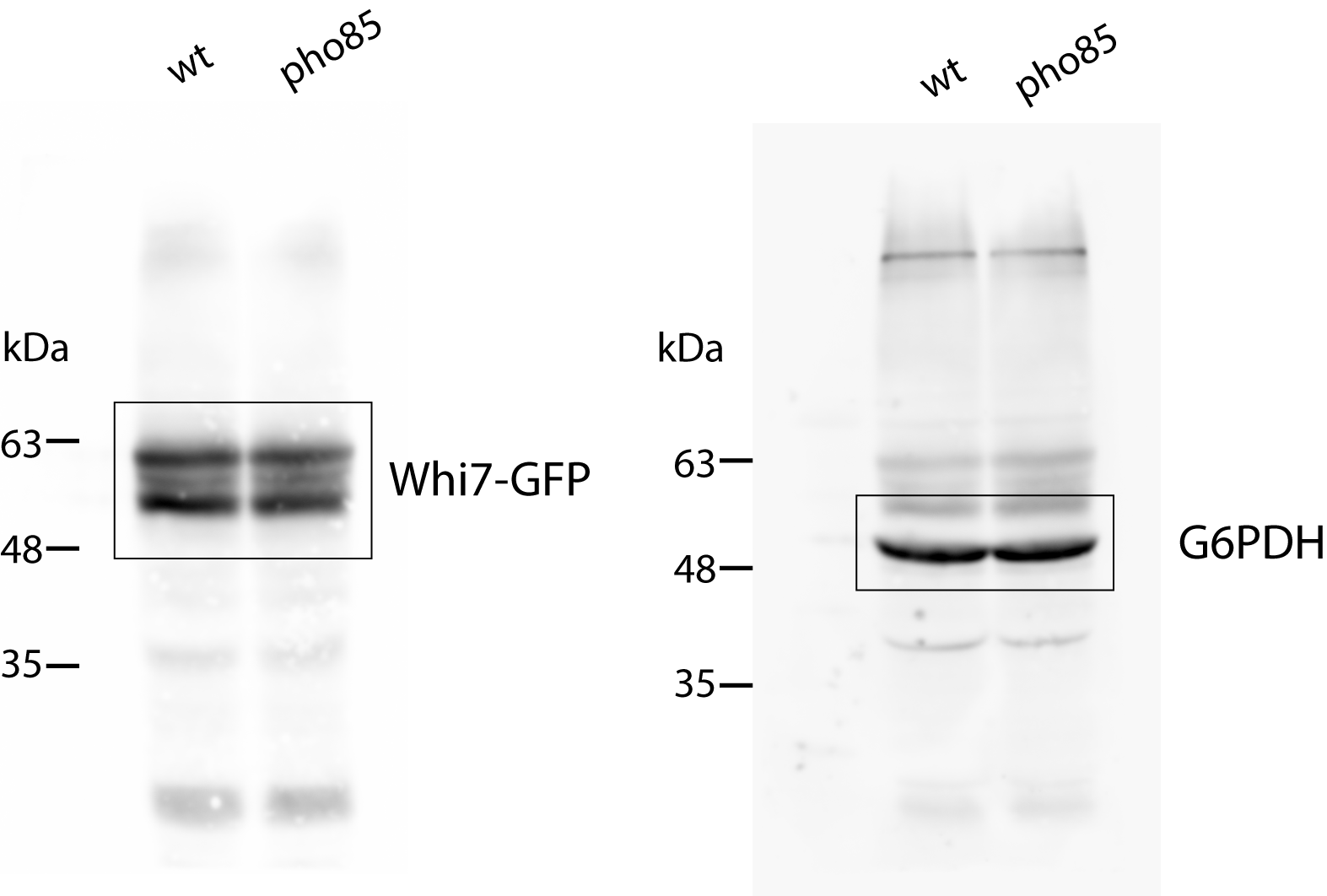

Supplement: Supplementary file 10 — Source Data Fig. 7 [file 44319_2023_49_MOESM10_ESM.zip › Figure 7/7B/WB_7B.tif]

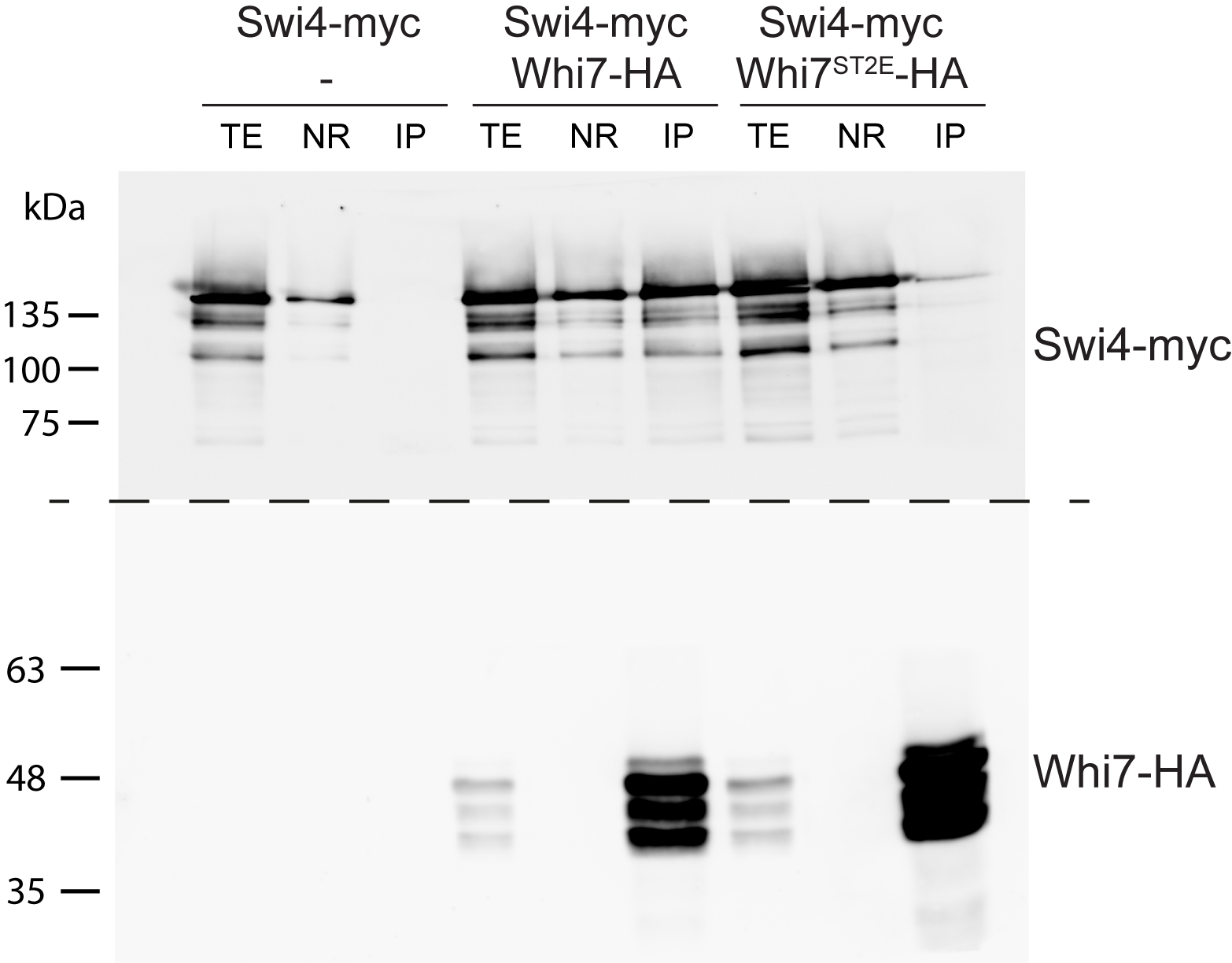

Supplement: Supplementary file 10 — Source Data Fig. 7 [file 44319_2023_49_MOESM10_ESM.zip › Figure 7/7G/WB_7G.tif]
