# Supplementary material for: The CDK Pho85 inhibits Whi7 Start repressor to promote cell cycle entry in budding yeast
Source: EMBO Rep. 2024 Jan 17;25(2):18. doi: 10.1038/s44319-023-00049-7 (PMC10897450; doi:10.1038/s44319-023-00049-7)
Supplement: Supplementary file 12 — Expanded View Figures [file 44319_2023_49_MOESM12_ESM.pdf]

Expanded View Figures

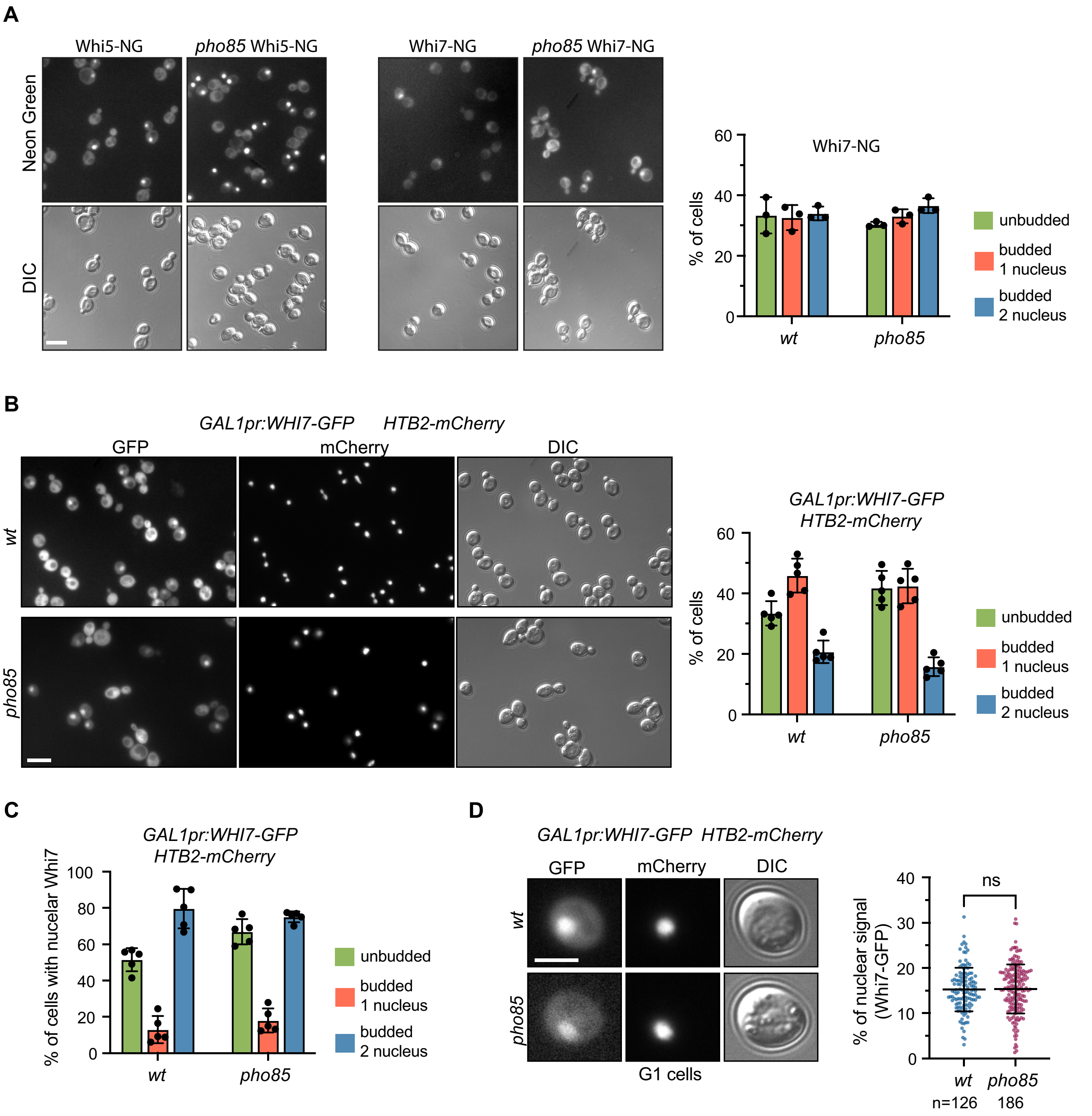

Figure EV1. The subcellular localization of Whi7 is not regulated by the CDK Pho85.

(**A**) Exponentially growing cells of the mNeonGreen-tagged Whi7 or Whi5 in wild-type (JCY2497 and JCY2495, respectively) and *pho85* (JCY2499 and JCY2501, respectively) strains were analyzed by conventional fluorescence microscopy. GFP and DIC channels are shown. Scale bar, 8 μm (left panel). Cell cycle distribution of wild-type and *pho85* cells carrying Whi7-NG ($n = 3$) (right panel). (**B**) Exponentially growing cells of the indicated strains (JCY2941 and JCY2951) were incubated with 1 nM β-estradiol for 1 h to obtain low Whi7-GFP induction. Cells were imaged by fluorescence microscopy. Scale bar, 8 μm (*left panel*) and the cell cycle distribution of wt and *pho85* cells carrying Whi7-GFP and Htb2-mCherry was sored (n = 5) (right panel). (**C**) In the same strains and conditions used in panel (**B**), the percentage of cells with nuclear Whi7 was scored in the different cell-cycle phases ($n = 5$). Htb2-mCherry was used as a nuclear marker. At least 200 cells were scored for each replicate and strain. (**D**) Cells of the same strains and conditions used in panel (**B**) were imaged by fluorescence microscopy and the levels of Whi7-GFP were determined in G1 cells. Total fluorescence intensity of Whi7-GFP was measured by segmentation of either the whole-cell in the DIC channel or the nuclear area in the mCherry channel and the percentage of nuclear signal was determined in G1 single cells. Scale bar 4 μm. Data information: In panels (**A–C**) data from the independent biological replicates are represented as mean ± s.d. In panel (**D**), mean ± s.d. of the single cells is shown. ns, $P > 0.05$, two-tailed unpaired *t*-test. In panels (**A**–C), n=number of biological replicates. In panel (**D**) n = number of cells.

© The Author(s)

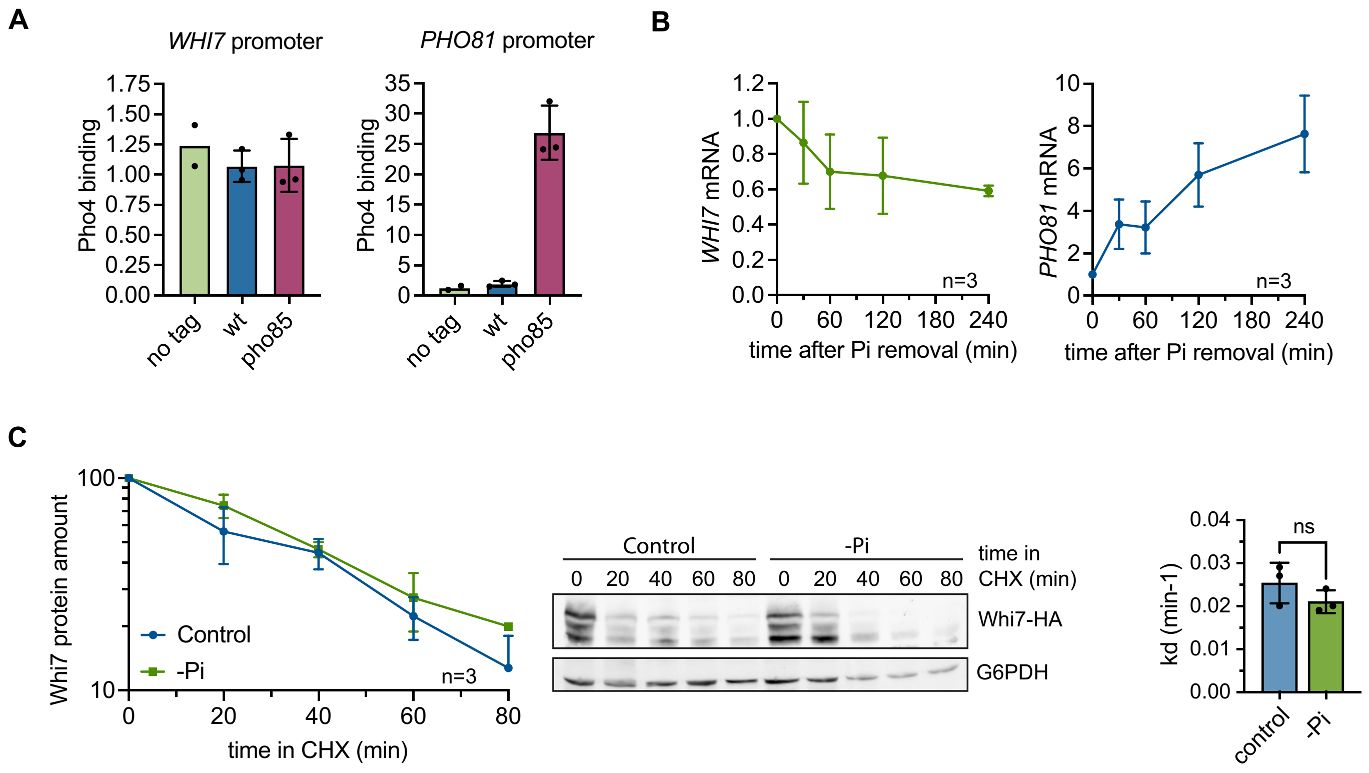

Figure EV2. Whi7 is not regulated by phosphate starvation conditions.

(**A**) Pho4 association to the *WHI7*, and *PHO81* gene promoters was analyzed by ChIP assays in wild-type (JCY2503) or *pho85* (JCY2505) cells expressing *PHO4-GFP* ($n = 2$–3). (**B**) The level of *WHI7* and *PHO81* mRNA relative to *ACT1* was analyzed by quantitative RT-PCR in wild-type (W303-1a) cells at the indicated times after phosphate (Pi) removal. Time 0 value is referred to as 1. (**C**) Whi7 protein stability was analyzed by translational shut-off with cycloheximide (CHX). Wild-type (JCY1728) cells were incubated for 3 h in either SC rich media (control) or SC low phosphate media (-Pi), CHX 100 μg/mL was added and Whi7 protein level was analyzed at the indicated times after the addition of CHX by western blot. Graphs represent the quantification of the western blots (left panel) and the degradation rate constant (right panel) of the indicated conditions. *G6PDH* is shown as loading control. Data information: In panels (**A**–**C**) data from the independent biological replicates are represented as mean ± s.d. ns, $P > 0.05$, two-tailed unpaired t-test. n = number of biological replicates.

 © The Author(s)

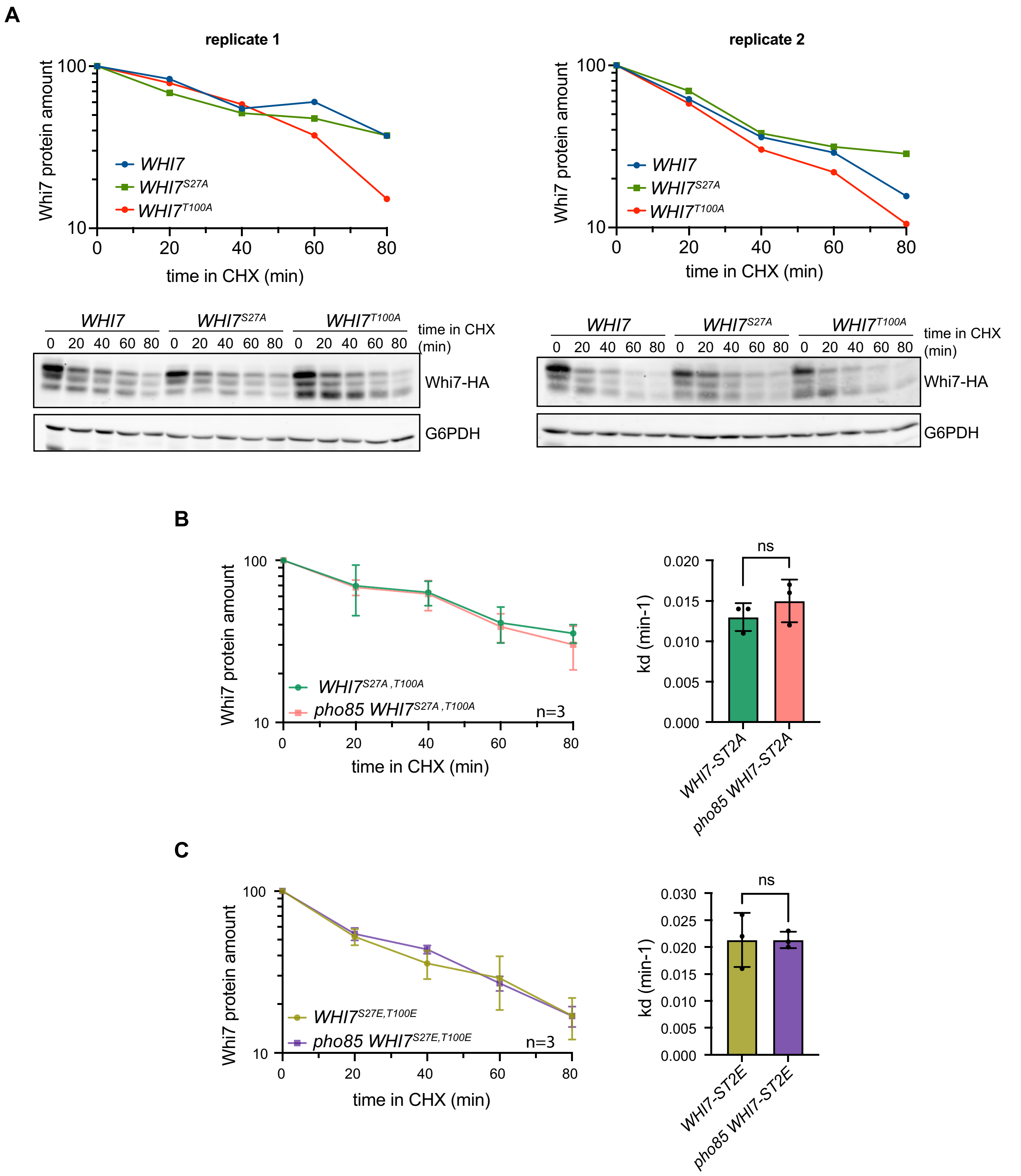

Figure EV3. Analysis of the Ser27 and Thr100 mutations on Whi7 protein stability.

(**A**) Whi7 protein stability was analyzed by translational shut-off with cycloheximide (CHX) in *whi7* cells (JCY1819) transformed with a centromeric plasmid expressing *WHI7*, *WHI7^{S27A}* or *WHI7^{T100A}*. Cells were incubated in the presence of CHX 100 µg/mL and Whi7 protein level was analyzed at the indicated times after the addition of CHX by western blot. *G6PDH* is shown as loading control. Graphs represent the quantification of two independent western blots ($n = 2$). (**B**,**C**) Whi7 protein stability was analyzed by translational shut-off as in panel (**A**) in wild-type and *pho85* cells carrying *WHI7$^{S27A, T100A}$* or *WHI7$^{S27E, T100E}$* expressed from a centromeric plasmid. Graphs represent the quantification of the western blots (left panel) and the degradation rate constant (right panel) of the indicated conditions. Data information: In panels (**B**,**C**) data from the independent biological replicates are represented as mean ± s.d. ns, $P > 0.05$, two-tailed unpaired *t*-test. n = number of biological replicates.

© The Author(s)

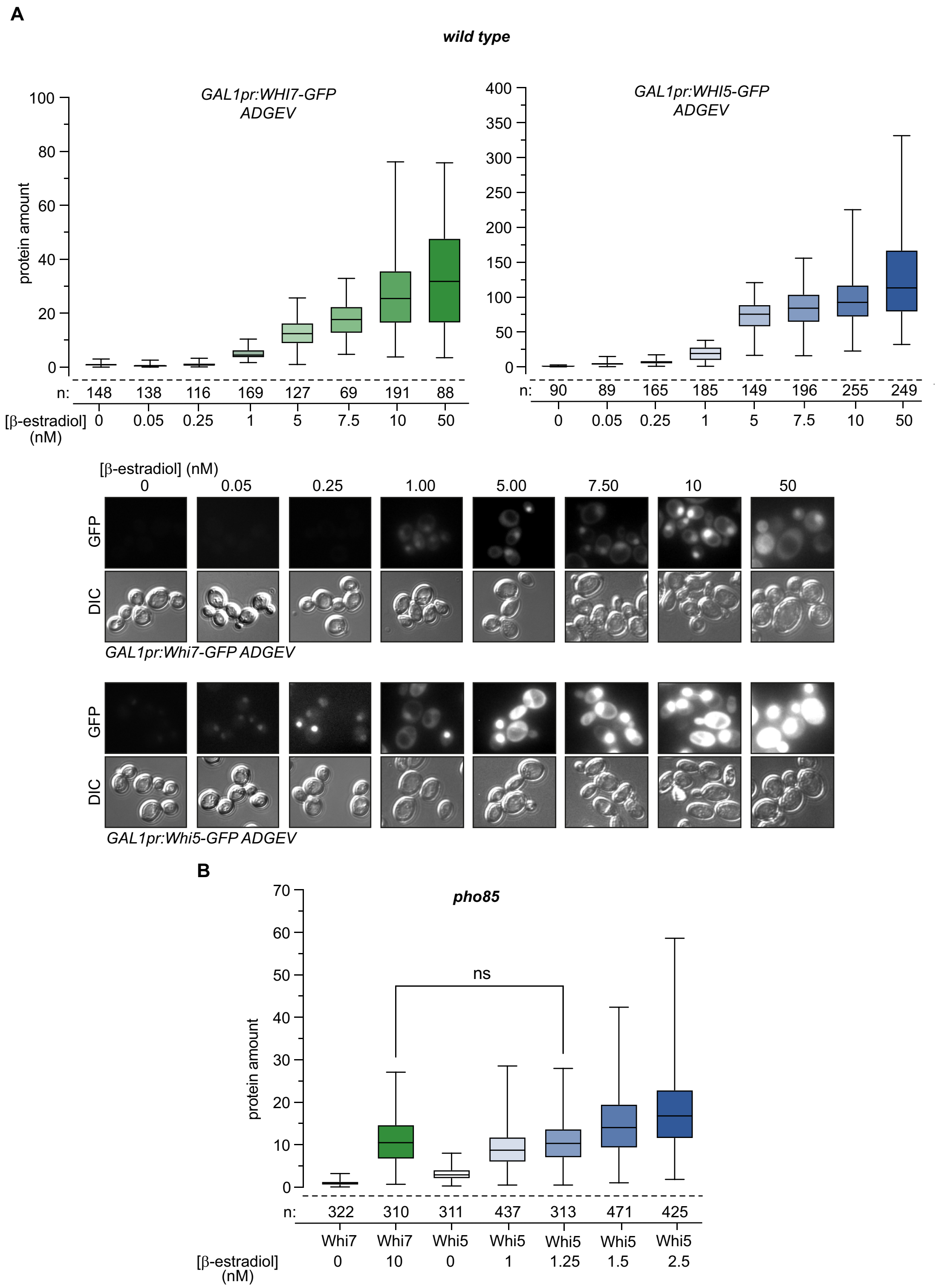

Figure EV4. Analysis of Whi7 and Whi5 protein levels at different β-estradiol concentrations.

(**A**) Quantification of total fluorescence in single wild-type cells expressing Whi7-GFP (JCY2446) (*left panel*) or Whi5-GFP (JCY2448) (right panel) induced over night with the indicated concentrations of β-estradiol. GFP fluorescence and DIC images of representative cells are shown. (**B**) Quantification of total fluorescence in single *pho85* mutant cells expressing Whi7-GFP (JCY2561) or Whi5-GFP (JCY2559) induced overnight with the indicated concentrations of c-estradiol. Graph represents data from one experiment. Data information: In panels (**A**,**B**), boxes include 50% of data points, the line represents the median, and whiskers extend to maximum and minimum values. To normalize fluorescence intensity, value 1 was given to the mean of Whi7-GFP 0 nM. ns, $P > 0.05$, two-tailed unpaired *t*-test. n = number of cells.

 © The Author(s)

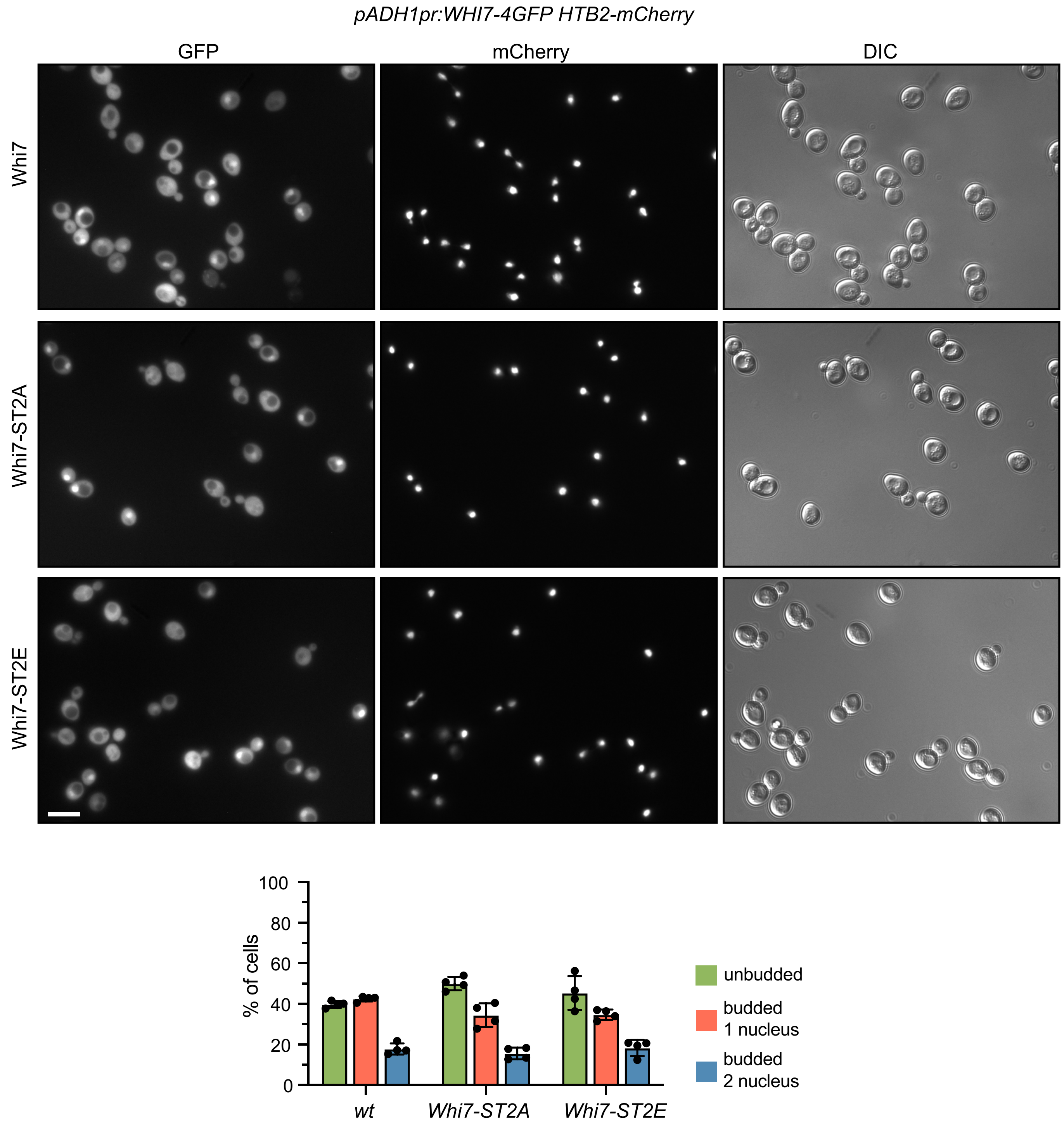

Figure EV5. The subcellular localization of Whi7 is not affected by the Ser27 and Thr100 mutations.

Exponentially growing cells of *whi7* cultures carrying Htb2-mCherry (JCY2981) transformed with a plasmid expressing *WHI7-4GFP*, *WHI7* $^{S27A,\ T100A}$*-4GFP* or *WHI7* $^{S27E,\ T100E}$*-4GFP* under the control of the *ADH1* promoter were imaged by fluorescence microscopy. GFP, mCherry and DIC channels are shown. Htb2-mCherry was used as nuclear marker. Scale bar 8 µm (upper panel). Cell cycle distribution of the imaged strains carrying the wild type and phosphomutant versions of Whi7 ($n = 4$). At least 200 cells were scored for each replicate and strain (lower panel). Data information: Data from the independent biological replicates are represented as mean ± s.d. n = number of biological replicates.
